# Design, Synthesis, and Biological Evaluation of PKD Inhibitors

**DOI:** 10.3390/pharmaceutics3020186

**Published:** 2011-04-21

**Authors:** Kara M. George, Marie-Céline Frantz, Karla Bravo-Altamirano, Courtney R. LaValle, Manuj Tandon, Stephanie Leimgruber, Elizabeth R. Sharlow, John S. Lazo, Q. Jane Wang, Peter Wipf

**Affiliations:** 1 Department of Chemistry, University of Pittsburgh, Pittsburgh, PA 15260, USA; 2 Department of Pharmacology and Chemical Biology, University of Pittsburgh, Pittsburgh, PA 15260, USA; 3 Department of Pharmacology, University of Virginia, Charlottesville, VA 22908, USA

**Keywords:** protein kinase D, small molecule inhibitor, benzothienothiazepinone, pyrimidines, CID755673, thiazepinothiophenopyrimidinone

## Abstract

Protein kinase D (PKD) belongs to a family of serine/threonine kinases that play an important role in basic cellular processes and are implicated in the pathogenesis of several diseases. Progress in our understanding of the biological functions of PKD has been limited due to the lack of a PKD-specific inhibitor. The benzoxoloazepinolone CID755673 was recently reported as the first potent and kinase-selective inhibitor for this enzyme. For structure-activity analysis purposes, a series of analogs was prepared and their *in vitro* inhibitory potency evaluated.

## 1. Introduction

Protein Kinase D (PKD) is a member of a novel family of serine/threonine kinases and diacylglycerol (DAG) receptors that have emerged as key regulators of many important cellular processes. The PKD family is now recognized as a subfamily of the Ca^2+^/calmodulin-dependent kinase superfamily. To date, three isoforms of PKD have been identified: PKD1 (formerly PKCμ) [1,2], PKD2 [3], and PKD3 (formerly PKCv) [4]. All three isoforms share a highly homologous sequence and a distinct structure that includes a catalytic domain, a pleckstrin homology (PH) domain, which mediates protein-protein interactions and PKD autoinhibition, and an *N*-terminal cysteine-rich DAG/phorbol ester binding domain (C1 domain). Both the activation of PKD and the regulatory mechanisms that control PKD activity have been well documented. PKD is activated by DAG-responsive protein kinase C (PKC) isoforms via transphosphorylation of the conserved Ser^744^ and Ser^748^ within the activation loop of PKD [5-7]. Subsequent autophosphorylation at multiple sites, including Ser^916^, confers full, sustained activity [8,9]. PKD is also subject to spatial regulation by DAG or phorbol esters and, as a result, is capable of shuttling between different subcellular compartments [10-12]. Accordingly, this canonical DAG/PKC/PKD pathway is crucial to fundamental PKD function in cells.

As a result of its central position in the signal transduction pathway, it is no surprise that PKD has been implicated in a variety of cellular processes, including cell proliferation, cell survival through oxidative stress-induced activation of nuclear factor-kappaB (NF-κB) signaling [13-15], gene expression by regulation of class IIa histone deacetylases (HDAC) [16-18], protein trafficking [19-21], cell motility [22,23], and immune responses [24-26]. These functions of PKD are known to impact many aspects of tumor biology, and extensive evidence indicates that PKD expression is dysregulated in multiple cancer types [27-30]. PKD also plays an active role in pathological processes such as cardiac hypertrophy [31,32], angiogenesis [33,34], and tumor cell proliferation and metastasis [35-37], making PKD an attractive therapeutic target for drug development [38].

**Figure 1.**
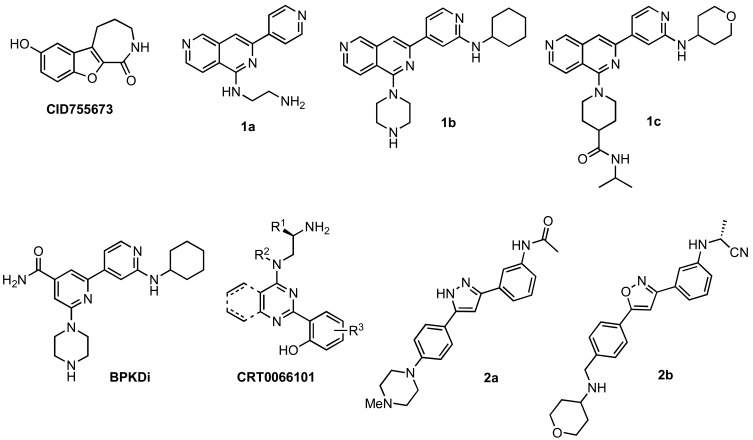
Recent PKD inhibitors reported in the research and patent literature.

Although extensive analysis of the role of PKD in biological processes has been hampered by the lack of both a three-dimensional structure and PKD specificity of early inhibitors (*i.e.* the isoquinoline sulfonamide H89 [39], staurosporine analogs [40,41], and resveratrol [42-44]), more potent and selective inhibitors have been reported recently [32,38,45-51]. The first breakthrough in this area came in 2008 with the identification and characterization of **CID755673** (Figure 1), a potent and PKD-selective kinase inhibitor [52]. **CID755673** was reported to inhibit all PKD isoforms with an IC50 of 200-300 nM, but showed specificity toward PKD over several related kinases [52]. Interestingly, **CID755673** was not competitive with ATP for enzyme inhibition, suggesting an alternate binding site on the enzyme, which may account for the selectivity for PKD compared to other protein kinases. This compound was also shown to effectively block PKD-mediated cell functions as well as the tumor-promoting functions of PKD in prostate cancer cells [52,53]. Despite the high specificity of **CID755673** and its potent *in vitro* inhibition of PKD, its cellular activity was relatively weak (EC50 = 11.8 μM) [52,53].

In addition to **CID755673**, promising ATP-competitive inhibitors continue to emerge in the literature [32,45,46,48-51]. The novel 2,6-naphthyridine **1a** (Figure 1) was identified by a high throughput screen (HTS) as a dual PKC/PKD inhibitor. Modification of this chemotype led to the orally available naphthyridine inhibitors **1b** and **1c** (Figure 1) [50]. Both **1b** and **1c** were able to block PKD phosphorylation and nuclear export of HDAC in the target tissue *in vivo*; however, their moderate kinase selectivity complicated data interpretation. In an effort to reduce the off-target effects and improve PKD selectivity, a series of amidobipyridyl-based analogs was generated [51]. From this series, **BPKDi** (Figure 1) was identified as a potent and selective inhibitor of all three isoforms of PKD with single digit nanomolar IC_50_ values and improved selectivity relative to **1b** [32,51]. Furthermore, **BPKDi** caused substantial inhibition of PKD1 signal-dependent phosphorylation and increased nuclear retention of class HDAC4 and HDAC5 in cardiomyocytes [32].

Another pan-PKD inhibitor, **CRT0066101** (Figure 1), was also reported to inhibit all isoforms of PKD with single-digit nanomolar IC_50_s. **CRT0066101** was shown to block cell proliferation, induce apoptosis, and reduce the viability of pancreatic cancer cells both *in vitro* and *in vivo*. As with the aforementioned ATP-competitive inhibitors, the most attractive features of **CRT0066101** are its reported orally availability and efficacy *in vivo* [46,48]. Lastly, preliminary studies of the structure-activity relationships (SAR) of a novel 3,5-diarylazole **2a** (Figure 1), which was identified in a HTS as a moderately potent kinase inhibitor, led to a series of promising benzamide analogs [49]. One analog in particular, **2b** (Figure 1), was found to inhibit all PKD isoforms with low nanomolar IC_50_s, while showing a 9- and 3-fold preference for PKD1 versus PKD2 and PKD3, respectively. Notably, **2b** shows high selectivity for PKD against a panel of other kinases, and pharmacokinetic studies in rats indicate that compound **2b** is orally available [49].

While all of these ATP-competitive, orally available PKD inhibitors represent valuable tools for further study of PKD signaling, their substantial off-target activity may likely be due to the high sequence homology near the hinge-binding regions of PKCs and PKDs. Recent evidence suggests additional targets of **CID755673** [54], but as this lead structure is not competitive with ATP for PKD inhibition, it can provide an orthogonal approach to gain further understanding of the structure and function of PKD. In an effort to enhance the selectivity and potency for potential *in vivo* applications, small molecule analogs of **CID755673** were generated by modification of the core structure as well as the side chains. We describe herein the complete SAR conducted thus far, which led to the discovery of a novel benzothienothiazepinone series. The improved PKD1 inhibitory activity of some of these analogs has already been highlighted in previous communications [53,55].

## 2. Results and Discussion

### 2.1. First generation SAR

Our investigations began with the chromenopyridine-based **CID797718**, a by-product of the synthesis of the parental compound, **CID755673** (Table 1). This compound was 10x less potent at PKD1 inhibition than **CID755673**. Efforts to improve the activity of **CID797718** by substitution of the phenolic hydroxyl group (Table 1, entries 1-3), *N*-alkylation (Table 1, entry 4), and *ortho-*halogenation (Table 1, entry 5) resulted in a complete loss of PKD1 activity. Consequently, the chromenopyridine scaffold was abandoned.

Modifications to the benzofuroazepinone scaffold present in **CID755673** proved to be more rewarding. The initial SAR analysis included modifications of the azepinone ring, substitution at the phenol and amide groups, and functionalization across the aryl moiety (Table 2). Changing the azepinone to either a 6- or 8-membered heterocycle resulted in a reduced inhibitory activity towards PKD1 (Table 2, entries 2–5). Substitutions of the phenolic hydroxyl group were not well tolerated either. Although both the methoxy and allyloxy substituents at that site showed moderate inhibitory activity towards PKD1 (Table 2, entries 6 and 7), the acetoxy and siloxy groups (Table 2, entries 8 and 9) showed poor inhibition and complete loss in activity, respectively. These results indicate that the aryl binding pocket of the protein may be restrictive in terms of both the size and the polarity of the aryl substituents. Similarly, alkylation/acylation of the amide or bioisosteric replacement with a dihydroimidazole resulted in weak or no PKD1 inhibition (Table 2, entries 10–14). These observations suggest that the amide functionality may provide crucial H-bonding interactions in the azepinone binding pocket that are required for optimal inhibitor-enzyme interactions.

Based on these results, subsequent synthetic analogs were biased to contain key features of the pharmacophore, *i.e.*, the 7-membered ring azepine, the monosubstituted lactam, and a small R^2^ substituent (Table 3). Derivatives bearing either a Cl or F atom at the R^1^ position retained activity against PKD1, but did not show improved potency relative to **CID755673** (Table 3, entries 1 and 2). In contrast, introduction of a Cl atom at either the R^3^ position, or both the R^1^ and R^3^ positions, provided inactive compounds (Table 3, entries 4 and 5). Interestingly, allyl and propenyl groups were well tolerated in this region, despite the aforementioned putative size restrictions of the aryl binding region (Table 3, entries 6 and 7). The inhibitory activity of these analogs also provides further support for a relatively hydrophobic aryl binding pocket. Finally, all modifications of the azepinone moiety at the 5 position (Z) yielded analogs with reduced inhibitory activity (Table 3, entries 8–12).

We next sought to investigate the replacement of the benzofuran core by a β-carboline skeleton (Table 4). Compound **kb-NB123-57** (Table 4, entry 2), which retains all functionality of **CID755673** except for the benzofuran oxygen being replaced with a nitrogen atom, exhibited comparable activity toward PKD1; however, there was no significant cellular activity. Variation of either the phenolic hydroxyl group or the lactam ring size in the β-carboline series did not provide any enhancement in activity (Table 4), and in cases where R^2^ was replaced with *O*-benzyl (Table 4, entries 3 and 4) or *N*-acyl (Table 4, entries 7 and 8), all inhibitory activity was lost. These modifications, accordingly, confirm that the 7-membered azepinone represents an optimal size and that the binding pocket of the protein is sterically demanding at the aryl binding region.

In the next SAR iteration, the benzofuran was replaced with a benzothiophene while the azepine was replaced by a thiazepine ring. Gratifyingly, both the benzothienothiazepinone **kb-NB142-70** and the methoxy analog **kb-NB165-09** showed a significance increase in potency toward PKD1, with IC_50_ values of 28.3 nM and 82.5 nM, respectively (Table 5, entries 2 and 3). This increased potency was further confirmed in cell-based assays, for which the cellular EC_50_ was lowered ca. 5-fold from 11.8 μM by **CID755673** to 2.2 μM and 3.1 μM by **kb-NB142-70** and **kb-NB165-09**, respectively. The remarkable activity of the methyl ether compared to the phenol indicates that a hydrogen bond donor at this position is not critical for activity. The lack of activity of the *O*-benzyl derivative for this series of analogs provides additional support for a sterically demanding aryl binding pocket.

### 2.2. Second generation SAR

**Figure 2.**
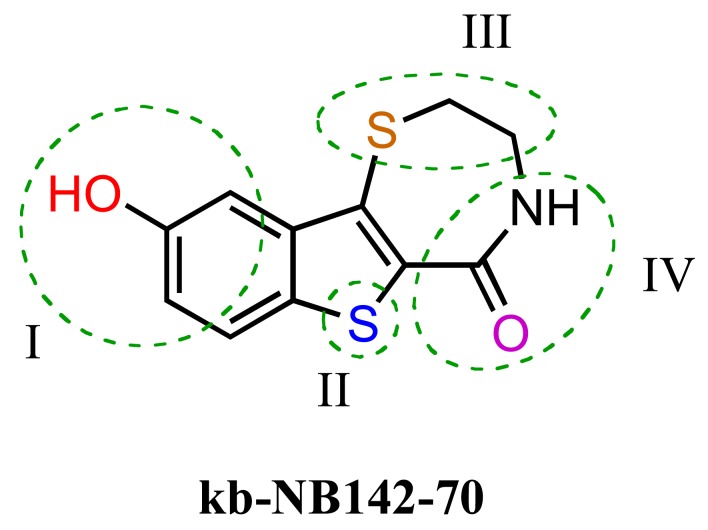
Major structural zones for SAR analysis based on **kb-NB142-70**.

The previous screening cycles led to the discovery of the benzothienothiazepinone **kb-NB142-70** as the most potent analog with an IC_50_ of 28.3 nM, which was an almost 7-fold improvement in potency over the parent compound, **CID755673**. Furthermore, we had gained considerable understanding of the SAR of the tricyclic inhibitor scaffold. Accordingly, we updated our pharmacophore model based on the structure of **kb-NB142-70**, and devised 4 major structural zones (Figure 2): zone I (aryl moiety), zone II (thiophene), zone III (thiazepinone), and zone IV (amide function). Each of these zones was subjected to selected structural modifications in order to elucidate essential activity relationships.

In zone I, the phenolic substituent and the substitution across the aryl moiety were modified (Table 6). Most of the zone I analogs were less active than the lead, **kb-NB142-70**. Specifically, substitutions at R^4^ were detrimental to *in vitro* activity (Table 6, entries 7–9), at least when R^2^ = H. In contrast, halogenation at R^1^ (Table 6, entries 5 and 6) and replacement of the phenolic hydroxy group with amine variants (Table 6, entries 1–4) were surprisingly well tolerated. Notably, the azide analog, **mcf292-08**, maintained a high inhibitory activity both *in vitro* and in cells, with IC_50_ values of 74.9 nM and 2.2 μM, respectively, thereby providing further support for the limited significance of a hydrogen bond donor at this position. The *ortho*-iodinated analog, **kb-NB165-31**, was also a potent inhibitor with an IC_50_ value of 114 nM; however, its cellular activity was 4-fold lower than that of **kb-NB142-70**. Masking the hydroxyl group of the lead **kb-NB142-70** was only slightly detrimental to the *in vitro* biological activity, as revealed by the methoxy analog **kb-NB165-09**. Moreover, preliminary results from an animal model suggest the glucuronidation of **kb-NB142-70** at the phenolic position to be a major metabolic pathway.

Therefore, the methoxy analogs were systematically developed in addition to the corresponding phenols in order to potentially circumvent a rapid excretion scenario *in vivo*. As part of our zone II and III modifications, the oxidation state of the benzothiophene sulfur atom, the size of the thiazepinone ring, and the oxidation and substitution of the thiazepinone ring sulfur atom were explored (Table 7). Sulfur oxidations in zones II and III provided analogs with reduced activity (Table 7, entries 1–3), while increasing the thiazepinone ring size by the addition of a methylene group had only minor effects on PKD1 inhibition for both the hydroxy and methoxy analogs (Table 7, entries 5 and 6). Additionally, the replacement of the thiazepinone ring sulfur atom with an oxygen resulted in a loss of inhibitory activity (Table 7, entries 7 and 8), suggesting high hydrophobicity and polarizability to be preferred in zone III. Attempts to exchange the thiazepinone ring sulfur atom with a nitrogen atom were met with preparative difficulties. The desired diazepinone ring could not be formed, and, as a result, an acyclic precursor was submitted for testing (Table 7, entry 9). The lack of activity of this analog suggests that the zone III binding pocket may require the rigidity of a ring system for optimal binding interactions.

Furthermore, modifications to zone IV, which included functional group interconversions and replacement of the amide moiety, did not enhance the inhibitory activity (Table 8). These results suggest that the unique hydrogen bond donor-acceptor capability of the amide is critical for protein interactions within the zone IV binding pocket.

The results from both the initial and the second generation SAR firmly established that the benzothienothiazepinones displayed superior PKD1 inhibition compared to the benzofuroazepinones. The most potent analog, **kb-NB142-70**, inhibited PKD1 with an IC_50_ of 28 nM, which is nearly a 7-fold improvement in potency compared to the parental compound **CID755673**. This improved analog also demonstrated increased inhibition of PMA-induced autophosphorylation of endogenous PKD1 in LNCaP prostate cancer cells with an EC_50_ of 2.2 μM, which represents a ca. 5-fold improvement in activity over **CID755673**, which has an EC_50_ of 11.8 μM.

However, despite the improvement of in vitro and cellular activities, *in vivo* studies revealed a short plasma half-life for this compound. In order to enhance the resistance of **kb-NB142-70** towards phase I and II metabolism, modifications to zone I were explored with the goal to install a more electron-deficient heteroarene ring (Table 9).

Gratifyingly, the methoxypyrimidine **kmg-NB4-23** exhibited an IC_50_ of 124 nM, which represents only a slight decrease in activity compared to that of the parent compound, **kb-NB165-09**. This result not only validates our design, but also suggests that the zone I binding pocket is tolerant to a decrease in electron density in the aryl region. Surprisingly, the hydroxy analog **kmg-NB4-69A** showed a significant loss in activity relative to the parental compound, **kb-NB142-70**. This effect can be attributed to the susceptibility of the C-4 position of the pyrimidine towards nucleophilic attack [56]; the heterocycle spontaneously degrades in protic solvents, resulting in reduced inhibitory activity towards PKD1. More hydrolytically stable pyrimidine analogs containing a substituent at the R^4^ position also exhibited a dramatic loss in activity, which is consistent with the general SAR results and provides additional support for a sterically limited zone I binding pocket. Unfortunately, the low aqueous solubility (<0.4 mg/mL even in the presence of lipophilic solubilizing agents) of **kmg-NB4-23** prevented the *in vivo* evaluation of this compound.

### 2.3. Synthetic chemistry

**Scheme 1.**
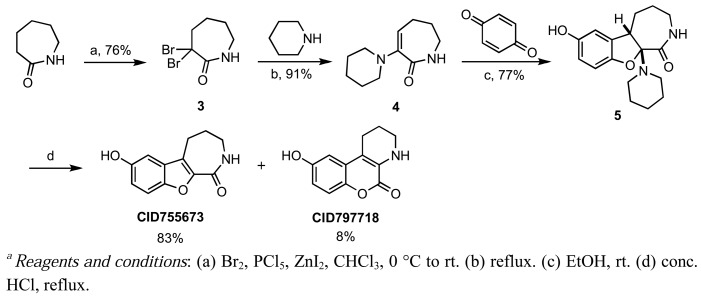
Synthesis of **CID755673** and **CID797718**.^*a*^

**CID755673** was initially formed in conjunction with **CID797718** in a synthetic route starting with commercially available ε-caprolactam, which was dibrominated at the α-position [57], and then treated with piperidine to afford the known α-oxolactam enamine **4** (Scheme 1) [58].

**Scheme 2.**
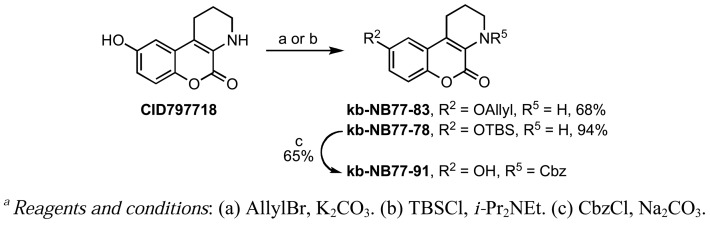
Functionalization of the chromenopyridine-based inhibitor **CID797718**.^*a*^

A Nenitzescu reaction between **4** and *para*-benzoquinone resulted in the formation of cyclic adduct **5**, which was in turn subjected to heating in aqueous acid [59] to provide the desired **CID755673**. Although **CID797718** was a byproduct of the synthesis of the target compound **CID755673**, it showed moderate activity and was therefore a desirable starting point for analog synthesis (Scheme 2). Functionalizations of this compound involved *O*- and *N*-alkylations, as well as chlorination across the aryl moiety. Allylation and TBS-protection of the phenolic hydroxy group of the parental compound, **CID797718**, provided the derivatives **kb-NB77-83** and **kb-NB77-78** in good yields. Treatment of **kb-NB77-78** with CbzCl under basic conditions afforded the desilylated carbamate **kb-NB77-91**.

**Scheme 3.**
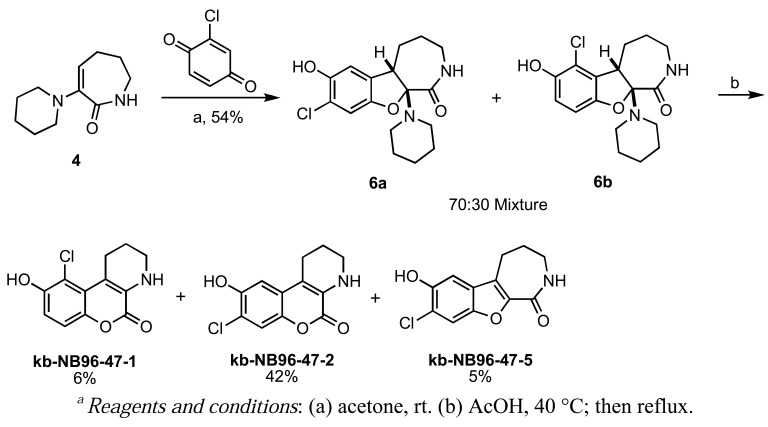
Synthesis of the chlorinated analogs of **CID797718** and **CID755673**.^*a*^

Introduction of a chlorine atom to the chromenopyridine scaffold was achieved by treating enamine **4** with 2-chloro-1,4-benzoquinone, followed by the same acid-promoted *cis-trans* isomerization-elimination as aforementioned in Scheme 1 (Scheme 3). The chlorinated analogs were isolated as a separable mixture of isomers. Unfortunately, all of the chromenopyridine-based analogs were inactive in the PKD1 inhibition assays, and therefore this scaffold was abandoned.

**Scheme 4.**
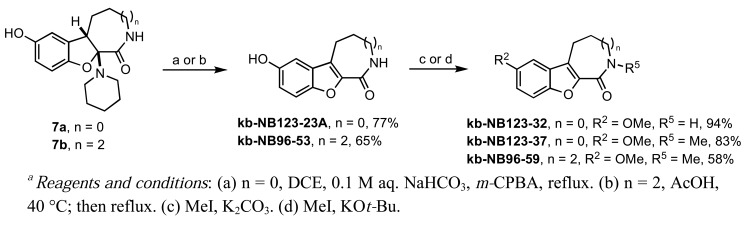
Functionalization of the 6- and 8-membered azepinone analogs.^*a*^

Our studies on the modification of **CID755673** began with the azepinone ring (Scheme 4). The syntheses of 6- and 8-membered ring analogs were carried out in an analogous manner to the sequence shown in Scheme 1. The acid-mediated piperidine elimination reaction of the 8-membered ring derivative **7b** proceeded with complete chemoselectivity toward the formation of the desired product **kb-NB96-53**. In contrast, treatment of **7a** with acetic acid provided the *N*-acetylated analog of the desired compound and the corresponding chromenopyrrole as the major products. Attempts to cleave the *N*-acetyl functionality to obtain the desired analog **kb-NB123-23A** were unsuccessful. Gratifyingly, subjecting **7a** to *m*CPBA at elevated temperatures afforded the desired 6-membered azepinone ring analog, **kb-123-23A**, in good yield. With the 6- and 8-membered ring analogs in hand, we next substituted the phenolic hydroxyl group and alkylated the azepinone amide nitrogen atom (Scheme 4).

Treatment of **kb-NB123-23A** with MeI and K_2_CO_3_ provided the *O*-methylated compound, **kb-NB123-32**, while use of a stronger base such as KO*t-*Bu gave the desired dimethylated analog, **kb-NB123-37**, in good yield. The 8-membered dimethylated product **kb-NB96-59** was obtained in a similar manner to **kb-NB123-37**, although only in modest yield.

Due to the lack of potency improvements for the 6- and 8-membered azepinone analogs, further modifications to the aryl region of the parental compound **CID755673** were explored. Functionalization of the phenol was achieved by treatment of **CID755675** with base in the presence of an electrophile (Table 10, entries 1–4).

The dimethylated compound **kb-NB96-04** and the diacylated compound **kb-NB123-45-1** were obtained in a similar manner by the use of either a stronger base (KO*t-*Bu) or additional equivalents of the desired electrophile, respectively (Table 10, entries 5 and 6). Modification of the aryl region also included additions of halogens (Table 10, entries 7–9). These derivatives could be obtained by the treatment of **CID755673** with either *N*-chlorosuccinimide in the case of the chlorinated analogs **kb-NB77-88** and **kb-NB96-43**, or Selectfluor^®^ in the case of the fluorinated analog **kb-NB96-21**.

**Scheme 5.**
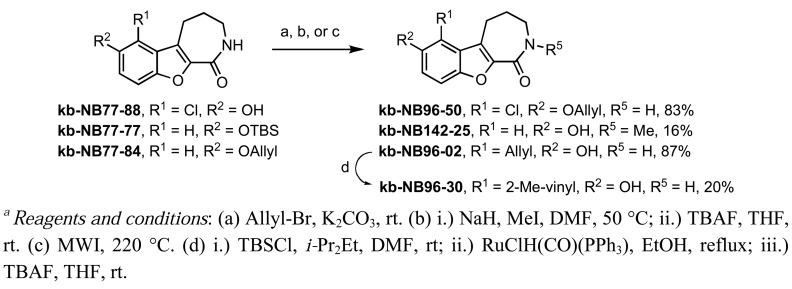
Functionalizations of the benzofuran-based analogs.^*a*^

Further modifications of these analogs were carried out in order to obtain a more complete SAR for the benzofuroazepinone series (Scheme 5). *O*-Allylation and *N*-methylation were performed on **kb-NB77-88** and **kb-NB77-77**, respectively. Additionally, the microwave-mediated Claisen rearrangement of the *O*-allylated-benzoxoloazepinolone **kb-NB77-84** provided the target compound **kb-NB96-02** with the allyl functionality at the R^1^ position. Protection of the phenol of **kb-NB96-02** with a silyl group provided a derivative which could be subjected to Ru-catalyzed olefin isomerization conditions. Subsequent TBS deprotection provided the isomerized product **kb-NB96-30** in modest yield over the three steps.

**Scheme 6.**
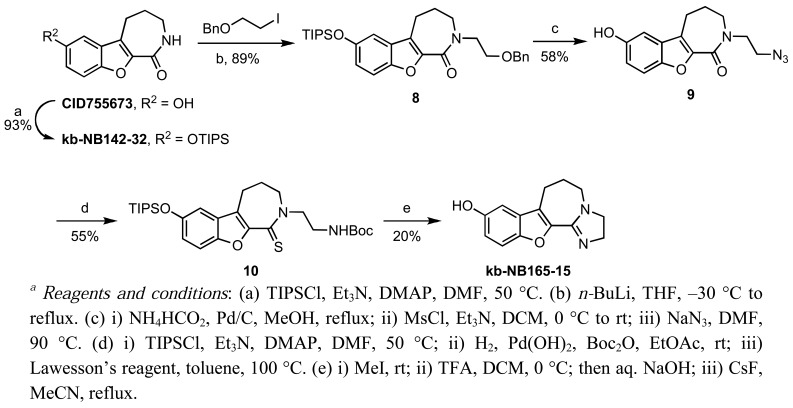
Isosteric replacement of the amide moiety of **CID755673** with an imidazoline.^*a*^

In addition to alkylations and acetylations of the amide moiety, we also synthesized **kb-NB165-15** to evaluate the effect of the isoelectronic replacement of the amide with an imidazoline ring (Scheme 6).

A TIPS protection of the parental compound **CID755673** followed by *N*-alkylation provided **8**. Debenzylation by transfer hydrogenation, mesylation of the primary alcohol, and displacement of the resulting mesylate with sodium azide afforded the desired alkyl azide **9** in good yield over the three steps. After protection of the phenol, the alkyl azide was reduced to the amine and protected, prior to treatment with Lawesson';s reagent to furnish thiolactam **10**. Cyclodethionation of thiolactam **10** [60] and subsequent TIPS deprotection delivered the desired imidazole containing analog, **kb-NB165-15**.

**Scheme 7.**
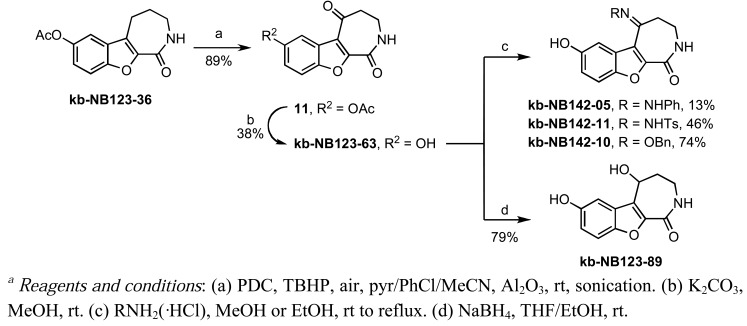
Fuctionalization of the azepinone moiety of **CID755673**.^*a*^

At this time, all modifications to the azepinone ring including altering the ring size, *N*-alkylations/acetylations, and isoelectronic replacements of the amide moiety had resulted in inhibitors with either reduced potency toward PKD1 or no inhibitory effect at all. Thus, we explored the introduction of functionality on the azepinone methylene groups to determine if this region of the molecule could be optimized further (Scheme 7).

The synthesis of these analogs began with the oxidation of the *O*-acetylated derivative **kb-NB123-36** with PDC-TBHP [61] in the presence of neutral alumina in order to install the desired ketone (**11**). The use of sonication proved to significantly improve both the yields and reproducibility of this oxidation. Subsequent acetyl deprotection in methanolic potassium carbonate solution provided **kb-NB123-63**. Access to further functionalized derivatives was achieved by acid-catalyzed condensation of **kb-NB123-63** with *O*-benzylhydroxylamine or *N*-substituted hydrazines (Scheme 7). The newly installed keto functionality was reduced using sodium borohydride to furnish the hydroxy derivative **kb-NB123-89**.

**Scheme 8.**
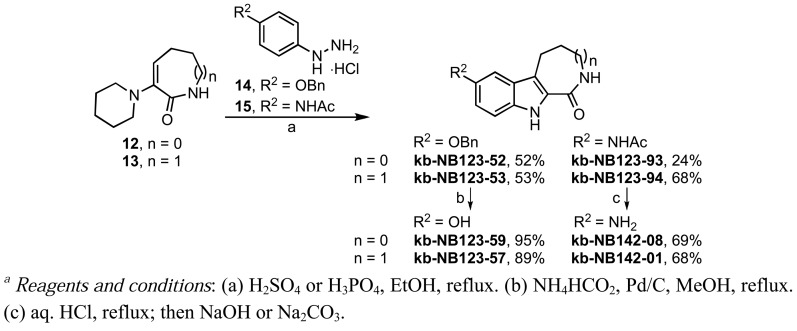
Replacement of the benzofuran scaffold by a β-carboline.^*a*^

We also investigated the replacement of the benzofuran core by a β-carboline scaffold (Scheme 8). β-Carbolines **kb-NB123-57** and **kb-NB123-59** were prepared from phenylhydrazine hydrochloride **14** [62,63] via a Fischer-like indole synthesis with the corresponding 6- and 7-membered ring α-keto lactams **12** and **13**. Lactams **12** and **13** were obtained *in situ* upon acid-catalyzed hydrolysis of the corresponding enamines [64]. Debenzylation of the resulting β-carbolines by transfer hydrogenation furnished the final phenol derivatives [63]. The aniline analogs, **kb-NB123-93** [65] and **kb-NB123-94**, were synthesized in a similar manner, starting from hydrazine hydrochloride **15** [66]. Subsequent cleavage of the acetyl group afforded the free amino β-carbolines (Scheme 8) [65].

**Scheme 9.**
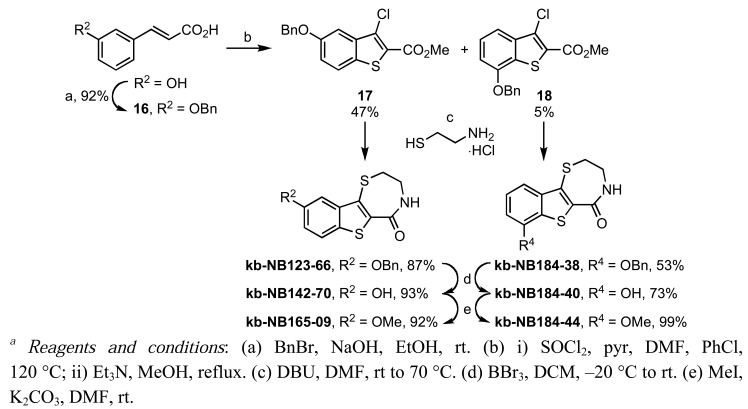
Synthesis of benzothienothiazepinone-based lead compound **kb-NB142-70** and zone I modifications.^*a*^

We also synthesized a thio variant of the benzofuroazepinone scaffold to study the effects of sulfur incorporation (Scheme 9).

This analog was constructed by a thionyl chloride-mediated Higa cyclization [67] of benzyl protected hydroxycinnamate **16** to a benzo[b]thiophene derived acid chloride, which was subsequently converted to the methyl ester **17**. Treatment of **17** with cysteamine hydrochloride in the presence of DBU furnished the benzyloxy-benzothienothiazepinone **kb-NB123-66** [68]. Deprotection of the aryl benzyl ether **kb-NB123-66** with boron tribromide provided **kb-NB142-70** in good yields, and subsequent *O*-methylation gave **kb-NB165-09** in excellent yield (Scheme 9). Gratifyingly, **kb-NB142-70** was identified as the most potent analog with an IC_50_ of 28.3 nM for PKD1, which represents nearly 7-fold higher potency over the parental benzofuroazepinone **CID755673**. We therefore dissected this lead compound into four major structural zones in order to further probe the SAR, as shown in Figure 2.

**Scheme 10.**
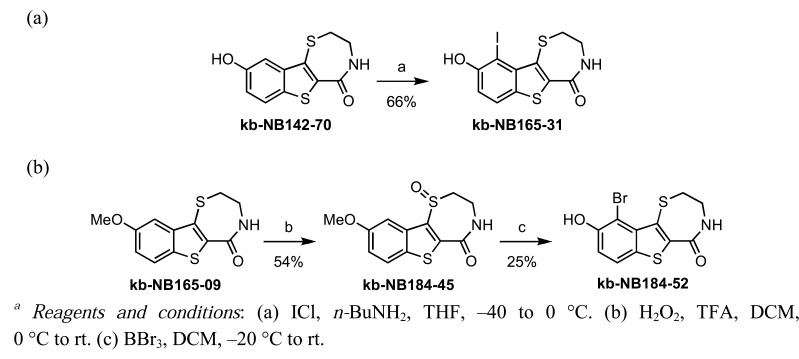
Zone I structural modifications.^*a*^

In zone I, we modified the substituent on the phenolic hydroxyl group and the position of this group (Scheme 9). The positional isomer **18** could be isolated as a byproduct from the thionyl chloride-mediated cyclization of **16**. Subsequent construction of the thiazepinone ring could be achieved via one-pot nucleophilic displacement-condensation reaction of **18** with cysteamine hydrochloride in a similar manner to the lead compound **kb-NB142-70**. Modification of this isomer by standard alkylation and protection-deprotection strategies provided the desired zone I analogs (Scheme 9). Zone I modifications also involved halogenations of the aryl moiety. Standard iodination conditions led to analog **kb-NB165-31** ([Scheme 10 (a)]).

**Scheme 11.**
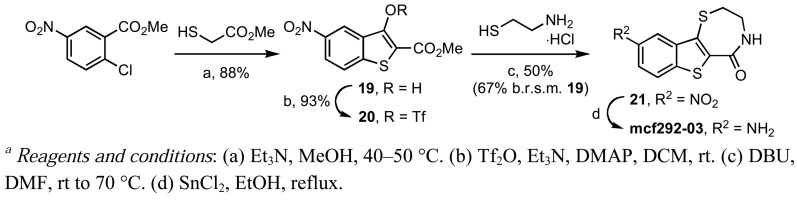
Synthesis of the aminobenzothienothiazepinone analog **mcf292-03**.^*a*^

In contrast, the brominated analog **kb-184-52** was synthesized from the corresponding sulfoxide **kb-NB184-45** upon treatment with BBr_3_ [Scheme 10 (b)]. Additional zone I modifications, which included replacement of the phenol group by nitrogen, required the development of an alternative route (Scheme 11 and Table 11). Nucleophilic aromatic substitution of methyl 2-chloro-5-nitrobenzoate by methyl thioglycolate anion followed by immediate Dieckman cyclization [63,69] afforded the benzothiophene precursor **19**. Cyclization of the corresponding triflate **20** with cysteamine hydrochloride provided the desired tricyclic core **21** in 50% yield (67% based on recovered starting material **19**, Scheme 11). Subsequent reduction of the nitro group furnished aniline **mcf292-03**, which was further functionalized by treatment with *t*-butyl nitrite and TMS-azide using Moses'; method [70] to yield the aryl azide, **mcf292-08** (Table 11, entry 1). The synthesis of the isothiocyanate **mcf292-05** was realized by subjecting **mcf292-03** to the modified Schotten-Baumann conditions reported by Nowick and co-workers [71] (Table 11, entry 2). Lastly, treatment of **mcf292-03** with chlororacetyl chloride in the presence of 2,6-lutidine provided the desired chloroacetyl analog **mcf292-09** in modest yield over two steps (Table 11, entry 3).

**Scheme 12.**
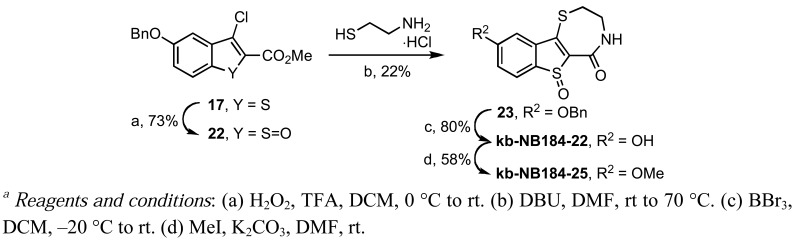
Zone II structural modifications.^*a*^

In our pursuit of zone II SAR, the sulfur ring atom of **17** was oxidized with trifluoroperacetic acid to the 3-chlorobenzo[b]thiophene-1-oxide **22** (Scheme 12). The vinylogous chloride **22** was then converted to the corresponding benzyloxybenzothienothiazepinone-6-oxide **23**, which upon benzyl deprotection and methylation provided the desired sulfoxide analogs, **kb-NB184-22** and **kb-NB184-25**, respectively (Scheme 12).

**Scheme 13.**
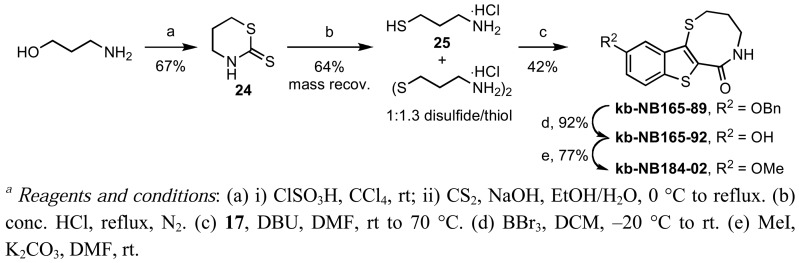
Zone III structural modifications.^*a*^

Zone III modifications included oxidations of the thiazepinone ring sulfur atom and variation of the thiazepinone ring size [Schemes 10 (b) and 13]. Selective oxidation of the thiazepinone ring sulfur atom could be achieved by treatment of **kb-NB165-09** with peracid to furnish **kb-NB184-45**, as shown in Scheme 10 (b). Preparation of the 8-membered thiazepinone ring analog began with the synthesis of **25** via the ring opening of thiazinane-thione **24** [72] (Scheme 13). Aminothiol **25** was isolated as a thiol/disulfide mixture and used directly in the aforementioned cyclocondensation-deprotection sequence to provide the desired 8-membered thiazepinone analogs (Scheme 13).

**Scheme 14.**
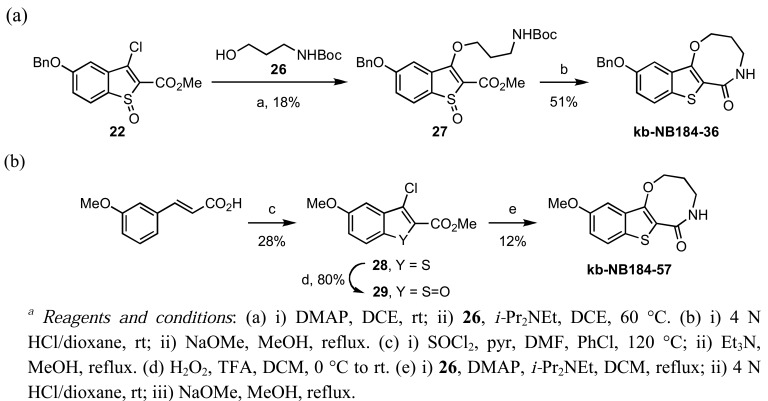
Zone III structural modifications.^*a*^

In an effort to further assess the SAR effects of modifying zone III, we investigated the synthesis of benzothiophene analogs linked to a three-carbon chain by an ether or amine function, instead of the thioether present in the lead compound. Key to accomplishing the synthesis of the ether analog **kb-NB184-36** was the use of the activated chloride **22**, which was obtained via oxidation of the benzothiophene sulfur to the corresponding sulfoxide according to Scheme 12. A DMAP-catalyzed nucleophilic displacement of the chlorine atom with alcohol **26** afforded the cyclization precursor **27**, which upon *N*-Boc deprotection and subsequent tandem cyclization-deoxygenation led to the benzyloxybenzothienooxazocinone analog **kb-NB184-36** [Scheme 14 (a) ]. The methoxy analog **kb-NB184-57** was synthesized in a similar manner [Scheme 14 (b) ].

**Scheme 15.**
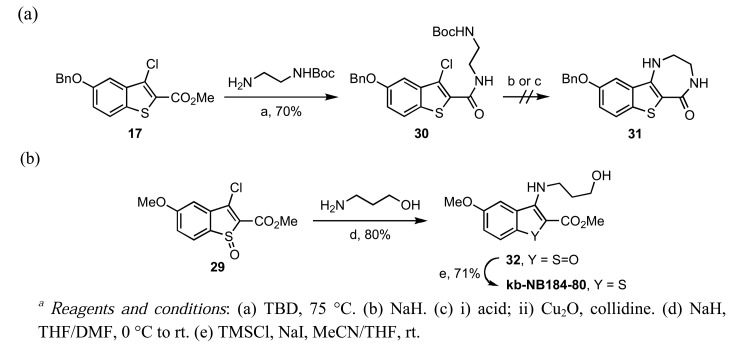
Zone III structural modifications.^*a*^

In an analogous manner, the synthesis of diazepinone analog **31** was attempted; however, precursor **30** failed to undergo cyclization under both base- and Cu-mediated conditions [Scheme 15 (a) ] [73]. Therefore, we opted for a nucleophilic displacement at the activated chlorine atom in **29** with aminopropanol, followed by a TMSI-mediated deoxygenation of **32** to provide **kb-NB184-80** [Scheme 15 (b) ]. Investigations regarding the use of these intermediates [Scheme 15 (b) ] as precursors for the desired analog **31** are still under investigation.

**Scheme 16.**
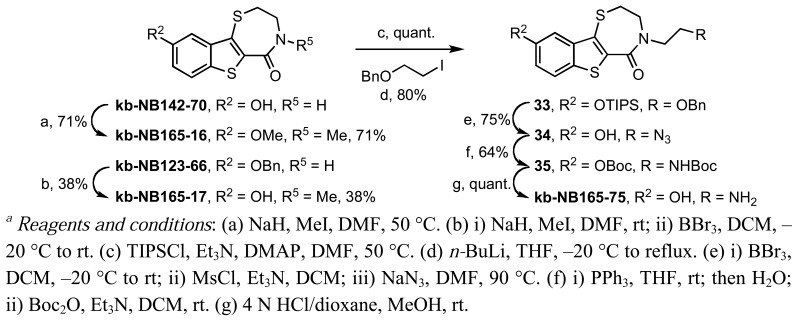
Zone IV structural modifications.^*a*^

**Scheme 17.**
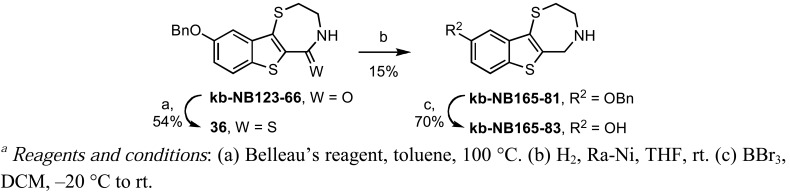
Zone IV structural modifications (continued).^*a*^

Finally, we explored structural modifications of zone IV by alkylation of the amide nitrogen and reduction of the amide (Schemes 16 and 17, respectively).

The *N*-methylated analog **kb-NB165-17** was prepared from the benzyloxybenzothiazepinone **kb-NB123-66** (Scheme 9) through an alkylation-deprotection sequence, while the dialkylated **kb-NB165-16** was obtained in one step by treatment with NaH and MeI. The synthesis of **kb-NB165-75** was accomplished via the *N*-alkylation of silyl protected **kb-NB142-70** with the corresponding alkyl iodide, followed by functional group interconversions and a final deprotection to furnish the desired analog **kb-NB165-75**. The amide reduction of thiolactam **36** proceeded poorly upon treatment with Raney-Nickel in THF due to a competitive cleavage of the other C-S bonds present in this system (Scheme 17). Nonetheless, we were able to isolate **kb-NB165-81** in low yields and debenzylate it to afford the desired phenol **kb-NB165-83** (Scheme 17).

**Scheme 18.**
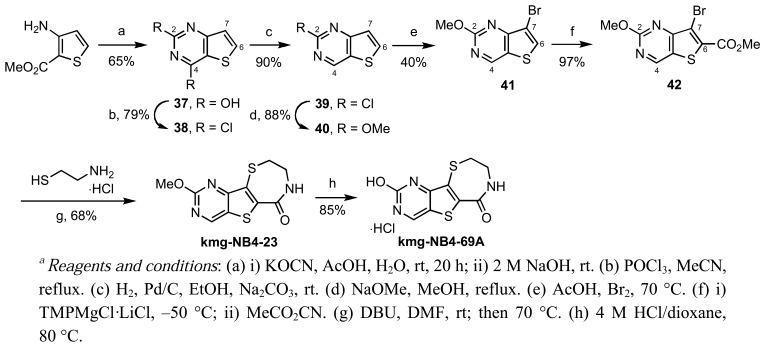
Synthesis of thiazepinothiophenopyrimidinone analogs.^*a*^

As mentioned in Section 2.2, preliminary *in vivo* studies revealed that the lead compound **kb-NB142-70** and the methoxy analog **kb-NB165-09** possessed a short plasma half-life (data not shown). Therefore, we further explored a zone I modification of **kb-NB142-70** to install a more electron-deficient pyrimidine moiety in place of the phenol ether, a known site of active phase I and II metabolism. The synthetic route to arrive at this new thiazepinothiophenopyrimidinone scaffold is summarized in Scheme 18.

**Figure 3.**
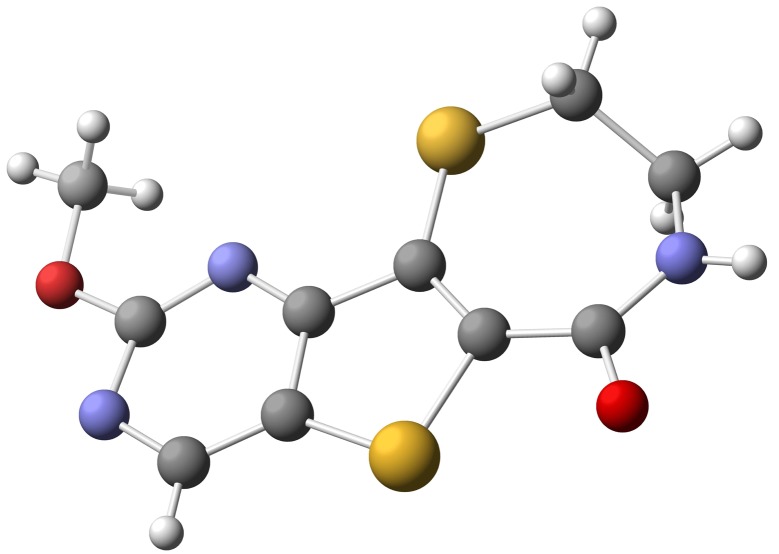
X-ray structure of **kmg-NB4-23**.

Starting with commercially available methyl 3-aminothiophene-2-carboxylate, formation of the pyrimidine moiety using potassium cyanate and chlorination with POCl_3_ provided dichloride **38** [74]. Regioselective palladium-catalyzed hydrogenolysis of **38** in the presence of Na_2_CO_3_ occurred exclusively at the C-4 position [75], and substitution of the remaining C-2 chloride with methoxide provided **40** in 79% yield over the two steps. Electrophilic bromination of **40** using bromine in acetic acid gave the desired C-7 bromo compound **41**. Functionalization at C-6 was accomplished via selective metalation and trapping with Mander';s reagent to provide the required cyclization precursor **42** [76]. Formation of the thiazepinone moiety was achieved by a one-pot nucleophilic displacement-condensation of **42** with cysteamine hydrochloride to provide the desired methoxypyrimidine **kmg-NB4-23** in good yield. The structure of **kmg-NB4-23** was confirmed by x-ray analysis (Figure 3; this structure has been deposited at the Cambridge Crystallographic Data Centre and allocated the deposition number CCDC 822403). When **kmg-NB4-23** was subjected to 4 M HCl in 1,4-dioxane, the desired hydroxypyrimidine **kmg-NB4-69A** was formed as the hydrochloride salt (Scheme 18).

**Scheme 19.**
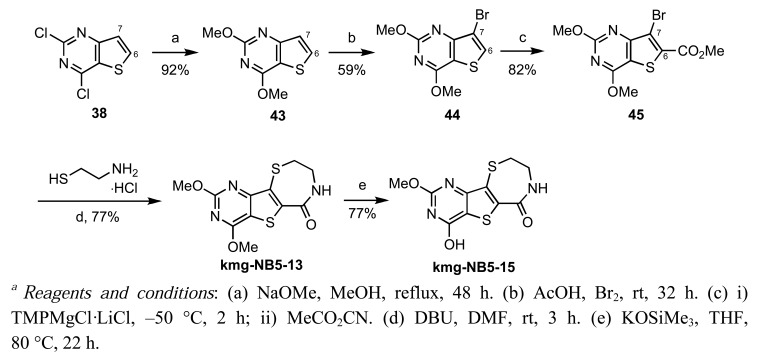
Synthesis of the thiazepinothiophenopyrimidinone analogs.^*a*^

Pyrimidine **kmg-NB4-23** is a potent nanomolar inhibitor of PKD, thus confirming the validity of our design. In contrast, **kmg-NB4-69A** had only weak inhibitory effect against PKD. This lack of activity is attributed to the instability of the compound towards nucleophilic addition of H_2_O at the C-4 position [56]. Efforts to stabilize the C-4 position led to the design of compounds **kmg-NB5-13** and **kmg-NB5-15** (Scheme 19).

These analogs were successfully synthesized in a similar manner to **kmg-NB4-23** and **kmg-NB4-69A** (Scheme 18). Unfortunately, preliminary biological data indicate an absence of inhibitory activity for both **kmg-NB5-13** and **kmg-NB5-15**.

## 3. Experimental Section

### 3.1. General

Melting points were determined on a Meltemp capillary melting point apparatus fitted with a Fluke 51 II digital thermometer. Infrared spectra were recorded on a Smiths IdentifyIR ATR spectrometer or a Perkin Elmer Spectrum 100 FT-IR spectrometer using the Universal ATR Sampling Accessory for both oil and solid compounds. ^1^H NMR and ^13^C NMR spectra were obtained on a Bruker Avance 300, 400 or 600 instrument at 300/75 MHz, 400/100 MHz or 600/150 MHz, respectively. Chemical shifts were reported in parts per million (ppm) as referenced to residual solvent. ^1^H NMR spectra are tabulated as follows: chemical shift, multiplicity (app = apparent, b = broad, s = singlet, d = doublet, t = triplet, q = quartet, quint = quintuplet, sext, = sextuplet, m = multiplet), number of protons, coupling constant(s). ^13^C NMR were obtained using a proton-decoupled pulse sequence and are tabulated by observed peak. Mass spectra were obtained on a Waters Autospec double focusing mass spectrometer (EI) or a Waters Q-Tof mass spectrometer (ESI), at the University of Pittsburgh Mass Spectrometry facility.

### 3.2. Characterization data for all final synthetic compounds

#### 9-Hydroxy-1,2,3,4-tetrahydro-chromeno[3,4-b]pyridin-5-one (**CID797718**)

Yield: 8%; m.p. (*i*-PrOH) 217–218 °C (lit. 213–216 °C) [59]; IR (ATR, neat) 3401, 3305 (br), 2937, 2879, 1662, 1583, 1449, 1342, 1219, 1184 cm^−1; 1^H-NMR (DMSO-*d*_6_, 600 MHz) δ 9.41 (s, 1 H), 7.09 (d, 1 H, *J* = 9.0 Hz), 6.74 (d, 1 H, *J* = 3.0 Hz), 6.66 (dd, 1 H, *J* = 9.0, 3.0 Hz), 5.91 (s, 1 H), 3.24–3.22 (m, 2 H), 2.59 (t, 2 H, *J* = 6.6 Hz), 1.90–1.85 (m, 2 H); ^13^C-NMR (DMSO-*d*_6_, 150 MHz) δ 158.1, 154.5, 140.9, 129.8, 122.9, 116.8, 114.8, 113.4, 106.4, 40.3, 21.6, 20.6; HRMS (ESI) *m/z* calcd for C_12_H_11_NO_3_ ([M+H]^+^) 218.0817, found 218.0802.

#### 9-Allyloxy-1,2,3,4-tetrahydrochromeno[3,4-b]pyridin-5-one (**kb-NB77-83**)

Yield: 68%; m.p. 90–91 °C; IR (ATR, neat) 3419, 3394, 2917, 2864, 1702, 1599, 1510, 1189 cm^−1; 1^H-NMR (CDCl_3_, 600 MHz) δ 7.20 (d, 1 H, *J* = 9.0 Hz), 6.86 (d, 1 H, *J* = 3.0 Hz), 6.84 (dd, 1 H, *J* = 9.0, 3.0 Hz), 6.11–6.04 (m, 1 H), 5.44 (d, 1 H, *J* = 16.8 Hz), 5.32 (d, 1 H, *J* = 10.2 Hz), 4.74 (bs, 1 H), 4.57 (d, 2 H, *J* = 5.4 Hz), 3.41–3.37 (m, 2 H), 2.69 (t, 2 H, *J* = 6.6 Hz), 2.08–2.03 (m, 2 H); ^13^C-NMR (CDCl_3_, 150 MHz) δ 158.8, 155.4, 142.5, 133.3, 129.7, 122.6, 118.0, 117.1, 115.9, 113.0, 106.0, 69.5, 40.6, 21.6, 20.8; HRMS (ESI) *m/z* calcd for C_15_H_15_NO_3_ ([M+H]^+^) 258.1130, found 258.1138.

#### 9-(tert-Butyldimethylsilyloxy)-1,2,3,4-tetrahydrochromeno[3,4-b]pyridin-5-one (**kb-NB77-78**)

Yield: 94%; m.p. 117–119 °C; IR (ATR, neat) 3418, 2929, 2856, 1721, 1699, 1602, 1508, 1430, 1251, 1172 cm^−1; 1^H-NMR (CDCl_3_, 600 MHz) δ 7.14 (d, 1 H, *J* = 9.0 Hz), 6.78 (d, 1 H, *J* = 3.0 Hz), 6.73 (dd, 1 H, *J* = 9.0, 3.0 Hz), 4.72 (bs, 1 H), 3.41–3.35 (m, 2 H), 2.67 (t, 2 H, *J* = 6.6 Hz), 2.09–2.02 (m, 2H), 1.00 (s, 9 H), 0.21 (s, 6 H); ^13^C-NMR (DMSO-*d_6_*, 150 MHz) δ 157.5, 151.7, 141.9, 129.5, 122.6, 117.1, 116.63, 116.57, 113.9, 111.0, 25.6 (3 C), 21.1, 20.0, 18.0, –4.5 (2 C); HRMS (ESI) *m/z* calcd for C_18_H_25_NO_3_Si ([M+H]^+^) 332.1682, found 332.1678.

#### Benzyl 9-hydroxy-5-oxo-1,2,3,4-tetrahydrochromeno[3,4-b]pyridine-4-carboxylate (**kb-NB77-91**)

Yield: 65%; m.p. 235–237 °C; IR (ATR, neat) 3887, 1724, 1679, 1404, 1308, 1261, 1160 cm^−1; 1^H-NMR (DMSO-*d_6_*, 600 MHz) δ 9.73 (s, 1 H), 7.38–7.33 (m, 4 H), 7.33–7.28 (m, 1 H), 7.23 (d, 1 H, *J* = 9.0 Hz), 6.98–6.95 (m, 2 H), 5.11 (s, 2 H), 3.30–3.50 (m, 2 H), 2.85 (t, 2 H, *J* = 6.6 Hz), 1.98–1.88 (m, 2 H); ^13^C-NMR (DMSO-*d_6_*, 150 MHz) δ 156.2, 154.03, 153.99, 144.8, 138.7, 136.1, 128.4 (2 C), 128.0, 127.7 (2 C), 125.0, 119.6, 118.4, 117.1, 108.5, 67.4, 44.2, 22.5, 22.1; HRMS (ESI) *m/z* calcd forC_20_H_17_NO_5_ ([M+Na]^+^) 374.1004, found 374.0991.

#### 10-Chloro-9-hydroxy-1,2,3,4-tetrahydrochromeno[3,4-b]pyridin-5-one (**kb-NB96-47-1**)

Yield: 6%; m.p. 211–213 °C; IR (ATR, neat) 3434, 3225 (br), 2971, 2906, 1666, 1589, 1509, 1339, 1235 cm^−1; 1^H-NMR (DMSO-*d_6_*, 600 MHz) δ 10.02 (s, 1 H), 7.10 (d, 1 H, *J* = 9.0 Hz), 6.85 (d, 1 H, *J* = 9.0 Hz), 6.24 (bs, 1 H), 3.25–3.20 (m, 2 H), 3.15 (t, 2 H, *J* = 6.6 Hz), 1.80–1.74 (m, 2 H); ^13^C-NMR (DMSO-*d_6_*, 150 MHz) 156.8, 151.0, 140.9, 130.4, 120.7, 115.5, 113.4, 113.1, 112.4, 26.8, 20.9; HRMS (EI) *m/z* calcd for C_12_H_10_ClNO_3_ (M^+^) 251.0349, found 251.0349.

#### 7-Hydroxy-2,3,4,5-tetrahydro-[1]benzoxolo[2,3-c]azepin-1-one (**CID755673**)

Yield: 83%; m.p. (*i-*PrOH) 245–247 °C (lit. 244–247 °C) [59]; IR (ATR, neat) 3187 (br), 3059, 2921, 1680, 1579, 1472, 1435, 1339, 1166 cm^−1; 1^H-NMR (DMSO-*d*_6_, 600 MHz) δ 9.36 (s, 1 H), 8.09 (t, 1 H, *J* = 4.8 Hz), 7.41 (d, 1 H, *J* = 9.0 Hz), 6.92 (d, 1 H, *J* = 2.4 Hz), 6.90 (dd, 1 H, *J* = 9.0, 2.4 Hz), 3.24 (dd, 2 H, *J* = 9, 4.8 Hz), 2.89 (t, 2 H, *J* = 6.6 Hz), 2.02–1.98 (m, 2 H); ^13^C-NMR (DMSO-*d*_6_, 150 MHz) δ 161.9, 153.9, 148.1, 144.3, 129.6, 123.5, 116.9, 112.4, 105.1, 41.2, 26.8, 24.3; HRMS (ESI) *m/z* calcd forC_12_H_11_NO_3_ ([M+H]^+^) 218.0817, found 218.0832.

#### 6-Hydroxy-3,4-dihydrobenzoxolo[2,3-c]pyridin-1(2H)-one (**kb-NB123-23A**)

Yield: 77%; m.p. 265–268 °C; IR (ATR, neat) 3404, 3158 (bs), 1661, 1588, 1479, 1451, 1339, 1228, 1208, 1184 cm^−1; 1^H-NMR (DMSO-*d_6_*, 600 MHz) δ 9.43 (s, 1 H), 7.74 (bs, 1 H), 7.47 (d, 1 H, *J* = 9.0 Hz), 6.98 (d, 1 H, *J* = 2.4 Hz), 6.91 (dd, 1 H, *J* = 9.0, 2.4 Hz), 3.50 (td, 2 H, *J* = 7.2, 2.4 Hz), 2.88 (t, 2 H, *J* = 7.2 Hz); ^13^C-NMR (DMSO-*d_6_*, 150 MHz) δ 159.4, 153.8, 149.0, 144.5, 126.6, 124.6, 116.4, 112.6, 105.1, 40.3, 20.0; HRMS (ESI) *m/z* calcd for C_11_H_9_NO_3_ ([M+H]^+^) 203.0582, found 203.0588.

#### 6-Methoxy-3,4-dihydrobenzoxolo[2,3-c]pyridin-1(2H)-one (**kb-NB123-32**) [77]

Yield: 94% m.p. 237–241 °C; IR (ATR, neat) 3197, 3088, 2895, 1672, 1585, 1480, 1432, 1329, 1214, 1192 cm^−1; 1^H-NMR (DMSO-*d_6_*, 600 MHz) δ 7.78 (s, 1 H), 7.58 (d, 1 H, *J* = 9.0 Hz), 7.26 (d, 1 H, *J* = 2.4 Hz), 7.06 (dd, 1 H, *J* = 9.0 Hz, 2.4 Hz), 3.81 (s, 3 H), 3.52 (td, 2 H, *J* = 7.2, 2.4 Hz), 2.94 (t, 2 H, *J* = 7.2 Hz); ^13^C-NMR (DMSO-*d_6_*, 150 MHz) δ 159.3, 156.0, 149.7, 144.7, 126.4, 125.0, 116.5, 112.9, 103.1, 55.7, 40.4, 20.1; HRMS (EI) *m/z* calcd for C_12_H_11_NO_3_ (M^+^) 217.0739, found 217.0743.

#### 8-Hydroxy-3,4,5,6-tetrahydrobenzoxolo[2,3-c]azocin-1(2H)-one (**kb-NB96-53**)

Yield: 77%; m.p. 225–232 °C; IR (ATR, neat) 3348, 3166 (br), 2927, 1639, 1578, 1465, 1433, 1216, 1153 cm^−1; 1^H-NMR (DMSO-*d_6_*, 600 MHz) δ 9.33 (s, 1 H), 7.98 (t, 1 H, *J* = 6.7 Hz), 7.36 (d, 1 H, *J* = 8.8 Hz), 6.87 (d, 1 H, *J* = 2.2 Hz), 6.85 (dd, 1 H, *J* = 8.8, 2.4 Hz), 3.32–3.27 (m, 2 H), 2.79–2.74 (m, 2 H), 1.92–1.86 (m, 2 H), 1.67–1.62 (m, 2 H); ^13^C-NMR (DMSO-*d_6_*, 150 MHz) δ 162.9, 153.5, 147.4, 142.6, 129.1, 122.2, 115.4, 111.7, 104.5, 30.2, 22.9, 20.5; HRMS (ESI) *m/z* calcd for C_13_H_13_NO_3_ ([M+Na]^+^) 254.0793, found 254.0773.

#### 8-Methoxy-2-methyl-3,4,5,6-tetrahydrobenzoxolo[2,3-c]azocin-1(2H)-one (**kb-NB96-59**)

Yield: 58%; m.p. 261–264 °C; IR (ATR, neat) 3168, 3038, 2928, 1651, 1581, 1478, 1435, 1210, 1152 cm^−1; 1^H-NMR (DMSO-*d_6_*, 600 MHz) δ 8.01 (s, 1 H), 7.47 (d, 1 H, *J* = 9.0 Hz), 7.12 (s, 1 H), 7.00 (d, 1 H, *J* = 9.0 Hz), 3.33–3.28 (m, 2 H), 3.80 (s, 3 H), 2.86–2.81 (m, 2 H), 1.93–1.86 (m, 2 H), 1.69–1.61 (m, 2 H); ^13^C-NMR (DMSO-*d_6_*, 150 MHz) δ 162.9, 155.8, 148.2, 142.9, 128.9, 122.7, 115.5, 112.1, 102.6, 55.7, 30.2, 23.0, 20.5; HRMS (ESI) *m/z* calcd for C_14_H_15_NO_3_ ([M+Na]^+^) 268.0950, found 268.0970.

#### 7-Methoxy-2,3,4,5-tetrahydro-[1]benzoxolo[2,3-c]azepin-1-one (**kb-NB77-56**)

Yield: 74%; m.p. 261–263 °C; IR (ATR, neat) 3200, 3063, 2936, 1642, 1580, 1474, 1434, 1207, 1163 cm^−1; 1^H-NMR (DMSO-*d_6_*, 600 MHz) δ 8.13 (t, 1 H, *J* = 4.8 Hz), 7.53 (d, 1 H, *J* = 9.0 Hz), 7.18 (d, 1 H, *J* = 2.4 Hz), 7.05 (dd, 1 H, *J* = 9.0, 2.4 Hz), 3.81 (s, 3 H), 3.27 (dd, 2 H, *J* = 9.0, 5.4 Hz), 2.96 (t, 2 H, *J* = 6.6 Hz), 2.06–2.00 (m, 2 H); ^13^C-NMR (DMSO-*d_6_*, 150 MHz) δ 161.4, 155.7, 148.4, 144.1, 129.0, 123.5, 116.4, 112.3, 102.8, 55.7, 40.8, 26.3, 24.0; HRMS (ESI) *m/z* calcd for C_13_H_14_NO_3_ ([M+H]^+^) 232.0974,found 232.0966.

#### 7-Allyloxy-2,3,4,5-tetrahydro-[1]benzoxolo[2,3-c]azepin-1-one (**kb-NB77-84**)

Yield: 69%; m.p. 198–200 °C; IR (ATR, neat) 3189, 3072, 2968, 2912, 1650, 1602, 1585, 1459, 1422, 1201, 1170 cm^−1^; 1H-NMR (DMSO-d6, 600 MHz) δ 8.13 (t, 1 H, *J* = 4.8 Hz), 7.53 (d, 1 H, *J* = 9.0 Hz), 7.20 (d, 1 H, *J* = 2.4 Hz), 7.08 (dd, 1 H, *J* = 9.0, 2.4 Hz), 6.11–6.04 (m, 1 H), 5.43 (dd, 1 H, *J* = 18, 1.8 Hz), 5.27 (dd, 1 H, *J* = 8.4, 1.8 Hz), 4.61 (d, 2 H, *J* = 5.22), 3.29–3.24 (m, 2 H), 2.94 (t, 2 H, *J* = 6.6 Hz), 2.06–1.98 (m, 2 H); ^13^C-NMR (DMSO-*d_6_*, 150 MHz) δ 161.4, 154.6, 148.4, 144.1, 133.8, 129.0, 123.5, 117.5, 116.8, 112.3, 104.1, 68.9, 40.8, 26.3, 23.9; HRMS (ESI) *m/z* calcd for C_15_H_15_NO_3_([M+Na]^+^) 280.0950, found 280.0959.

#### 7-Acetoxy-2,3,4,5-tetrahydro-[1]benzoxolo[2,3-c]azepin-1-one (**kb-NB123-36**)

Yield: 91%; m.p. 196–197 °C; IR (ATR, neat) 3193, 3083, 2935, 1750, 1663, 1583, 1207, 1157, 1063 cm^−1; 1^H-NMR (DMSO-*d_6_*, 600 MHz) δ 8.21 (bs, 1 H), 7.66 (d, 1 H, *J* = 9.0 Hz), 7.47 (d, 1 H, *J* = 2.4 Hz), 7.22 (dd, 1 H, *J* = 9.0, 2.4 Hz), 3.27 (dd, 2 H, *J* = 8.4, 4.8 Hz), 2.94 (t, 2 H, *J* = 6.6 Hz), 2.29 (s, 3 H), 2.06–1.99 (m, 2 H); ^13^C-NMR (DMSO-*d_6_*, 150 MHz) δ 169.7, 161.2, 151.0, 146.3, 144.6, 129.0, 123.5, 122.6, 113.9, 112.3, 40.8, 26.3, 23.8, 20.9; HRMS (EI) *m/z* calcd for C_14_H_13_NO_4_ (M^+^) 259.0845, found 259.0850.

#### 7-(tert-Butyldimethylsilyloxy)-2,3,4,5-tetrahydro-[1]benzoxolo[2,3-c]azepin-1-one (**kb-NB77-77**)

Yield: 91%; m.p. 209–212 °C; IR (ATR, neat) 3194, 3085, 2952, 2927, 1655, 1579, 1467, 1252, 1202, 1170 cm^−1; 1^H-NMR (DMSO-*d_6_*, 600 MHz) δ 8.13 (bs, 1 H), 7.50 (d, 1 H, *J* = 9.0 Hz), 7.06 (d, 1 H, *J* = 1.8 Hz), 6.96 (dd, 1 H, *J* = 8.4, 2.4 Hz), 3.28–3.23 (m, 2 H), 2.93 (t, 2 H, *J* = 6.6 Hz), 2.04–1.97 (m, 2 H), 0.97 (s, 9 H), 0.19 (s, 6 H); ^13^C-NMR (DMSO-*d_6_*, 150 MHz) δ 161.3, 151.0 148.8, 144.2, 129.3, 123.3, 120.4, 112.2, 110.3, 40.8, 26.p3, 25.6 (3 C), 23.9, 18.0, –4.6 (2 C); HRMS (ESI) *m/z* calcdfor C_18_H_25_NO_3_Si ([M+Na]^+^) 354.1501, found 354.1472.

#### 6-Methoxy-2-methyl-3,4-dihydrobenzoxolo[2,3-c]pyridin-1(2H)-one (**kb-NB123-37**)

Yield: 83%; m.p. 168–172 °C; IR (ATR, neat) 2920, 1661, 1602, 1485, 1454, 1329, 1212, 1179 cm^−1; 1^H-NMR (DMSO-*d_6_*, 600 MHz) δ 7.57 (d, 1 H, *J* = 9.0 Hz), 7.25 (d, 1 H, *J* = 2.4 Hz), 7.05 (dd, 1 H, *J* = 9.0, 2.4 Hz), 3.81 (s, 3 H), 3.69 (t, 2 H, *J* = 7.2 Hz), 3.01 (t, 2 H, *J* = 7.2 Hz), 2.98 (s, 3 H); ^13^C-NMR (DMSO-*d_6_*, 150 MHz) δ 158.5, 156.0, 149.9, 144.5, 123.8, 116.4, 112.9, 103.0, 55.7, 48.6, 33.5, 19.3; HRMS (EI) *m/z* calcd for C_13_H_13_NO_3_ (M^+^) 231.0895, found 231.0899.

#### 7-Hydroxy-2-methyl-2,3,4,5-tetrahydro-[1]benzoxolo[2,3-c]azepin-1-one (**kb-NB142-25**)

Yield: 36%; m.p. 282–284 °C; IR (ATR, neat) 3186, 1615, 1577, 1452, 1407, 1364, 1325, 1187 cm^−1; 1^H-NMR (DMSO-*d_6_*, 600 MHz) δ 9.36 (s, 1 H), 7.41 (d, 1 H, *J* = 9.0 Hz), 6.92 (d, 1 H, *J* = 2.4 Hz), 6.90 (dd, 1 H, *J* = 9.0, 2.4 Hz), 3.52–3.49 (m, 2 H), 3.06 (s, 3 H), 2.86 (t, 2 H, *J* = 6.6 Hz), 2.03–2.09 (m, 2 H); ^13^C-NMR (DMSO-*d_6_*, 150 MHz) δ 160.0, 153.5, 147.5, 144.0, 129.0, 122.4, 116.3, 111.9, 104.6, 49.3, 36.2, 25.5, 23.3; HRMS (EI) *m/z* calcd for C_13_H_13_NO_3_ (M^+^) 231.0895, found 231.0899.

#### 7-Methoxy-2-methyl-2,3,4,5-tetrahydro-[1]benzoxolo[2,3-c]azepin-1-one (**kb-NB96-04**)

Yield: 34%; m.p. 150–151 °C; IR (ATR, neat) 3008, 2919, 1629, 1579, 1478, 1440, 1428, 1217, 1177 cm^−1; 1^H-NMR (DMSO-*d_6_*, 600 MHz) δ 7.53 (d, 1 H, *J* = 9.0 Hz), 7.16 (d, 1 H, *J* = 2.4 Hz), 7.05 (dd, 1 H, *J* = 9.0, 2.4 Hz), 3.81 (s, 3 H), 3.54–3.50 (m, 2 H), 3.07 (s, 3 H), 2.93 (t, 2 H, *J* = 6.6 Hz), 2.11–2.05 (m, 2 H); ^13^C-NMR (DMSO-*d_6_*, 150 MHz) δ 159.9, 155.7, 148.3, 144.3, 128.9, 122.9, 116.3, 112.3, 102.7, 55.7, 49.3, 36.2, 25.5, 23.4; HRMS (ESI) *m/z* calcd for C_14_H_15_NO_3_ ([M+Na]^+^) 268.0950, found 268.0935.

#### 2-Acetyl-1-oxo-2,3,4,5-tetrahydro-[1]benzoxolo[2,3-c]azepin-7-yl acetate (**kb-NB123-45-1**)

Yield: 33%; m.p. 155–157 °C; IR (ATR, neat) 3069, 2949, 1753, 1699, 1669, 1570, 1402, 1363, 1213, 1168 cm^−1; 1^H-NMR (DMSO-*d_6_*, 600 MHz) δ 7.75 (d, 1 H, *J* = 9.0 Hz), 7.61 (s, 1 H, *J* = 2.4 Hz), 7.31 (dd, 1 H, *J* = 9.0, 2.4 Hz), 3.96–3.92 (m, 2 H), 3.03 (t, 2 H, *J* = 6.0 Hz), 2.45 (s, 3 H), 2.30 (s, 3 H), 2.08–2.03 (m, 2 H); ^13^C-NMR (DMSO-*d_6_*, 150 MHz) δ 171.3, 169.6, 162.3, 151.9, 146.6, 144.5, 128.4, 127.1, 123.1, 114.5, 112.7, 40.9, 26.2, 25.1, 21.6, 20.9; HRMS (EI) *m/z* calcd for C_16_H_15_NO_5_(M^+^) 301.0950, found 301.0964.

#### 9-Hydroxy-3,5,6,7-tetrahydro-2H-benzoxolo[2,3-c]imidazo[1,2-a]azepine (**kb-NB165-15**)

Yield: 40%; m.p. 230–232 °C (dec., brown), 268–270 °C (dec., melts); IR (ATR, neat) 2918, 2871, 2528, 1624, 1590, 1550, 1445, 1392, 1285, 1195 cm^−1; 1^H-NMR (CD_3_OD, 600 MHz) δ 7.34 (d, 1 H, *J* = 9.6 Hz), 6.92–6.90 (m, 2 H), 3.81 (t, 2 H, *J* = 10.2 Hz), 3.63 (t, 2 H, *J* = 10.2 Hz), 3.44–3.41 (t, 2 H, *J* = 4.8 Hz), 2.95 (t, 2 H, *J* = 6.0 Hz), 2.22–2.17 (m, 2 H); ^13^C-NMR (CD_3_OD, 150 MHz) δ 159.8, 155.6, 150.3, 142.4, 130.6, 125.8, 117.9, 112.8, 105.6, 55.0, 52.0, 49.4, 26.5, 25.1; HRMS (EI) *m/z* calcd for C_14_H_14_N_2_O_2_ (M^+^) 242.1055, found 242.1052.

#### 6-Chloro-7-hydroxy-2,3,4,5-tetrahydro-[1]benzoxolo[2,3-c]azepin-1-one (**kb-NB77-88**)

Yield: 86%; m.p. >300 °C; IR (ATR, neat) 3886, 3036 (br), 2928, 1629, 1566, 1428, 1340, 1174 cm^−1; 1^H-NMR (DMSO-*d_6_*, 600 MHz) δ 10.0 (s, 1 H), 8.22 (t, 1 H, *J* = 4.8 Hz), 7.43 (d, 1 H, *J* = 9.0 Hz), 7.10 (d, 1 H, *J* = 9.0 Hz), 3.26–3.20 (m, 4 H), 2.04–1.98 (m, 2 H); ^13^C-NMR (DMSO-*d_6_*, 150 MHz) δ 161.1, 149.5, 147.9, 144.6, 126.0, 123.4, 116.8, 111.4, 110.8, 40.2, 27.0, 26.3; HRMS (ESI) *m/z* calcd for C_12_H_10_ClNO_3_ ([M+Na]^+^), 274.0247, found 274.0226.

#### 6-Fluoro-7-hydroxy-2,3,4,5-tetrahydro-[1]benzoxolo[2,3-c]azepin-1-one (**kb-NB96-21**)

Yield: 29%; m.p. >300 °C; IR (ATR, neat) 3197 (br), 2925, 2385, 1625, 1577, 1477, 1437, 1345, 1032 cm^−1; 1^H-NMR (DMSO-*d_6_*, 600 MHz) δ 9.67 (bs, 1 H), 8.19 (t, 1 H, *J* = 4.2 Hz), 7.26 (d, 1 H, *J* = 9.0 Hz), 7.08 (dd, 1 H, *J* = 8.6 Hz, *J_HF_* = 8.6 Hz), 3.25 (dd, 2 H, *J* = 8.4, 4.8 Hz), 3.09 (t, 2 H, *J* = 6.6 Hz), 2.05–1.97 (m, 2 H); ^13^C-NMR (DMSO-*d_6_*, 150 MHz) δ 161.1, 147.8 (d, *J_CF_* = 7.5 Hz), 144.3 (d, *J_CF_* = 244 Hz), 143.9, 139.8 (d, *J_CF_* = 10.3 Hz), 121.8 (d, *J_CF_* = 3.5 Hz), 118.3 (d, *J_CF_* = 4.5 Hz), 118.1, 107.4 (d, *J_CF_* = 4.2 Hz), 40.5, 26.7, 25.4; ^19^F NMR (DMSO-*d_6_*, 400 MHz) δ –149.09 (d, *J_FH_* = 8.4 Hz); HRMS (ESI) *m/z* calcd for C_12_H_10_FNO_3_ ([M+Na]^+^) 258.0542, found 258.0566.

#### 7-Allyloxy-6-chloro-2,3,4,5-tetrahydro-[1]benzoxolo[2,3-c]azepin-1-one (**kb-NB96-50**)

Yield: 83%; representative experimental data: m.p. 192–194 °C; IR (ATR, neat) 3200, 3075, 2928, 1674, 1650, 1573, 1464, 1422, 1259, 1173, 1065 cm^−1; 1^H-NMR (DMSO-*d_6_*, 600 MHz) δ 8.28 (t, 1 H, *J* = 4.2 Hz), 7.58 (d, 1 H, *J* = 9.0 Hz), 7.33 (d, 1 H, *J* = 9.0 Hz), 6.10–6.02 (m, 1 H), 5.44 (dd, 1 H, *J* = 17.4, 1.8 Hz), 5.28 (dd, 1 H, *J* = 9, 1.8 Hz), 4.69 (s, 2 H), 3.28–3.21 (m, 4 H), 2.05–1.97 (m, 2 H); ^13^C-NMR (DMSO-*d_6_*, 150 MHz) δ 160.9, 150.1, 148.9, 145.1, 133.5, 126.2, 123.4, 117.7, 115.2, 114.6, 110.8, 70.4, 30.7, 26.9, 26.3; HRMS (ESI) *m/z* calcd for C_15_H_14_ClNO_3_ ([M+Na]^+^) 314.0560, found 314.0540.

#### 8-Chloro-7-hydroxy-2,3,4,5-tetrahydro-[1]benzoxolo[2,3-c]azepin-1-one (**kb-NB96-47-5**)

Yield: 5%; representative experimental data: m.p. 327–332 °C; IR (ATR, neat) 3293, 3196 (br), 2938, 1652, 1578, 1464, 1437, 1233, 1141 cm^−1; 1^H-NMR (DMSO-*d_6_*, 600 MHz) δ 10.12 (s, 1 H), 8.14 (bs, 1 H), 7.73 (s, 1 H), 7.11 (s, 1 H), 3.28–3.22 (m, 2 H), 2.89 (t, *J* = 6.6 Hz, 2 H), 2.03–1.98 (m, 2 H); ^13^C-NMR (DMSO-*d_6_*, 150 MHz) δ161.2, 149.4, 147.0, 144.3, 128.0, 123.0, 120.9, 112.7, 105.7, 40.7, 26.3, 23.7; HRMS (ESI) *m/z* calcd for C_12_H_10_ClNO_3_ ([M+Na]^+^) 274.0247, found 274.0224.

#### 6,8-Dichloro-7-hydroxy-2,3,4,5-tetrahydro-[1]benzoxolo[2,3-c]azepin-1-one (**kb-NB96-43**)

Yield: 73%; m.p. 298–301 °C; ^1^H-NMR (DMSO-*d_6_*, 600 MHz) δ 9.91 (s, 1 H), 8.29 (bs, 1 H), 7.83 (s, 1 H), 3.25–3.20 (m, 4 H), 2.04–1.99 (m, 2 H); ^13^C-NMR (DMSO-*d_6_*, 150 MHz) δ 160.8, 147.2, 145.7, 145.1, 125.1, 123.4, 123.1, 114.8, 111.7, 40.1, 26.9, 26.1; IR (ATR, neat) 3377, 3077 (br), 2969, 1643, 1568, 1428, 1327, 1233, 1171 cm^–1^; HRMS (ESI) *m/z* calcd for C_12_H_9_Cl_2_NO_3_ ([M+Na]^+^), 307.9857, found 307.9882.

#### 6-Allyl-7-hydroxy-2,3,4,5-tetrahydro-[1]benzoxolo[2,3-c]azepin-1-one (**kb-NB96-02**)

Yield: 87%; m.p. 255–257 °C; IR (ATR, neat) 3174 (br), 3056, 2920, 1641, 1571, 1476, 1426, 1358, 1343, 1272, 1118 cm^−1; 1^H-NMR (DMSO-*d_6_*, 600 MHz) δ 9.19 (s, 1 H), 8.12 (t, 1 H, *J* = 4.8 Hz), 7.28 (d, 1 H, *J* = 9.0 Hz), 6.98 (d, 1 H, *J* = 9.0 Hz), 6.00 (ddt, 1 H, *J* = 17.4, 10.2, 5.4 Hz,), 4.97 (dd, 1 H, *J* = 10.2, 1.2 Hz), 4.79 (dd, 1 H, *J* = 17.4, 1.2 Hz), 3.64 (d, 2 H, *J* = 5.4 Hz), 3.20 (dd, 2 H, *J* = 9.0, 5.4 Hz), 3.14 (t, 2 H, *J* = 6.6 Hz), 2.02–1.95 (m, 2 H); ^13^C-NMR (DMSO-*d_6_*, 150 MHz) δ 161.6, 150.9, 148.1, 143.8, 137.5, 127.5, 124.0, 118.4, 116.1, 114.8, 109.7, 40.3, 29.1, 27.3, 25.9; HRMS (ESI) *m/z* calcd forC_15_H_15_NO_3_ ([M+Na]^+^), 280.0950, found 280.0960.

#### 6-[(E)-2-(Prop-1-enyl) ]-7-hydroxy-2,3,4,5-tetrahydro-[1]benzoxolo[2,3-c]azepin-1-one (**kb-NB96-30**)

Yield: 37%; m.p. >300 °C; IR (ATR, neat) 3147 (br), 2908, 1644, 1568, 1477, 1417, 1245, 1165 cm^−1; 1^H-NMR (CD_3_OD, 600 MHz) δ 7.23 (d, 1 H, *J* = 9.0 Hz), 6.97 (d, 1 H, *J* = 9.0 Hz), 6.69 (dd, 1 H, *J* = 15.6, 1.2 Hz), 6.22 (dq, 1 H, *J* = 15.6, 6.6 Hz), 3.40–3.37 (m, 2 H), 3.15 (t, 2 H, *J* = 6.6 Hz), 2.15–2.10 (m, 2 H), 1.95 (dd, 3 H, *J* = 6.6, 1.8 Hz); ^13^C-NMR (CD_3_OD, 150 MHz) δ 165.2, 152.0, 150.5, 144.4, 133.8, 128.0, 127.9, 124.8, 120.7, 118.2, 111.0, 42.4, 29.5, 28.3, 19.3; HRMS (ESI) *m/z* calcd for C_15_H_15_NO_3_ ([M+Na]^+^) 280.0950, found 280.0950.

#### 7-Hydroxy-3,4-dihydro-[1]benzoxolo[2,3-c]azepine-1,5(2H)-dione (**kb-NB123-63**)

Yield: 38%; m.p. >300 °C; IR (ATR, neat) 3189 (br), 3072, 2919, 1674, 1645, 1544, 1461, 1349, 1262, 1223 cm^−1; 1^H-NMR (DMSO-*d_6_*, 600 MHz) δ 8.87 (t, 1 H, *J* = 4.8 Hz), 7.58 (d, 1 H, *J* = 9.0 Hz), 7.57 (d, 1 H, *J* = 2.4 Hz), 6.97 (dd, 1 H, *J* = 9.0, 2.4 Hz), 3.51–3.46 (m, 2 H), 2.93–2.87 (m, 2 H); ^13^C-NMR (DMSO-*d_6_*, 150 MHz) δ 195.7, 159.8, 155.4, 151.3, 147.9, 125.5, 119.1, 117.1, 112.5, 107.2, 44.1, 36.0; HRMS (EI) *m/z* calcd for C_12_H_9_NO_4_ (M^+^) 231.0532, found 231.0530.

#### 5,7-Dihydroxy-2,3,4,5-tetrahydro-[1]benzoxolo[2,3-c]azepin-1-one (**kb-NB123-89**)

Yield: 79%; m.p. 280–282 °C; IR (ATR, neat) 3189 (br), 3074, 2918, 1646, 1577, 1455, 1436, 1337, 1178 cm^−1; 1^H-NMR (CD_3_OD, 600 MHz) δ 7.39 (d, 1 H, *J* = 9.0 Hz), 7.23 (d, 1 H, *J* = 2.4 Hz), 6.97 (dd, 1 H, *J* = 9.0, 2.4 Hz), 5.14 (d, 1 H, *J* = 4.2 Hz), 3.58 (dd, 1 H, *J* = 15.0, 10.2 Hz), 3.37–3.32 (m, 1 H), 2.32–2.26 (m, 1 H), 2.21–2.14 (m, 1 H); ^13^C-NMR (CD_3_OD, 150 MHz) δ 164.4, 155.0, 150.4, 144.2, 130.1, 127.5, 118.2, 113.0, 107.3, 64.6, 37.3, 37.1; HRMS (EI) *m/z* calcd for C_12_H_11_NO_4_ (M^+^) 233.0688, found 233.0691.

#### 7-Hydroxy-5-(2-phenylhydrazono)-2,3,4,5-tetrahydro-[1]benzoxolo[2,3-c]azepin-1-one (**kb-NB142-05**)

Yield: 13%; m.p. 234–236 °C; IR (ATR, neat) 3218 (br), 2924, 1643, 1600, 1553, 1447, 1341, 1250, 1144 cm^−1; 1^H-NMR (CD_3_OD, 600 MHz) δ 7.93 (d, 1 H, *J* = 2.4 Hz), 7.41 (d, 1 H, *J* = 8.4 Hz), 7.34–7.27 (m, 4 H), 7.01 (dd, 1 H, *J* = 9, 2.4 Hz), 6.87 (tt, 1 H, *J* = 6.0, 1.2 Hz), 3.60–3.57 (m, 2 H), 3.00–2.97 (m, 2 H); ^13^C-NMR (CD_3_OD, 150 MHz) δ 165.7, 155.4, 150.8, 146.8, 144.6, 139.2, 130.2 (2 C), 127.5, 124.8, 121.3, 118.1, 114.5 (2 C), 112.8, 110.6, 39.0, 32.4; HRMS (EI) *m/z* calcd for C_18_H_15_N_3_O_3_ (M^+^) 321.1113, found 321.1110.

#### 7-Hydroxy-5-[2-{(4-methylphenyl)sulfony}hydrazono]-2,3,4,5-tetrahydro-[1]benzoxolo[2,3-c]azepin-1-one (**kb-NB142-11**)

Yield: 46%; m.p. 220–224 °C; IR (ATR, neat) 3212 (br), 1648, 1559, 1448, 1334, 1161 cm^−1; 1^H-NMR (DMSO-*d_6_*, 600 MHz) δ 10.69 (s, 1 H), 9.53 (s, 1 H), 8.48 (bs, 1 H), 7.91 (d, 2 H, *J* = 7.2 Hz), 7.55 (s, 1 H), 7.45 (d, 1 H, *J* = 9.0 Hz), 7.35 (d, 2 H, *J* = 7.80 Hz),6.94 (d, 1 H, *J* = 8.4 Hz), 3.33–3.28 (m, 2 H), 2.76–2.83 (m, 2 H), 2.33 (s, 3 H); ^13^C-NMR (CD_3_OD, 150 MHz) δ 164.6, 155.8, 151.2, 150.6, 147.0, 145.6, 137.3, 130.6 (2 C), 129.2 (2 C), 127.0, 122.3, 118.3, 112.7,110.8, 38.6, 32.9, 21.5; HRMS (EI) *m/z* calcd for C_19_H_17_N_3_O_5_S (M^+^), 422.0787, found 422.0817.

#### 5-(Benzyloxyimino)-7-hydroxy-2,3,4,5-tetrahydro-[1]benzoxolo[2,3-c]azepin-1-one (**kb-NB142-10**)

Yield: 74%; m.p. 103–110 °C; IR (ATR, neat) 3217 (br), 2925, 1648, 1552, 1467, 1448, 1351, 1334, 1209, 1185 cm^−1; 1^H-NMR (CD_3_OD, 600 MHz) δ 7.66 (t, 1 H, *J* = 1.8 Hz), 7.47 (dd, 2 H, *J* = 7.8, 0.6 Hz), 7.41 (dd, 1 H, *J* = 9.0, 1.2 Hz), 7.37 (dt, 2 H, *J* = 7.8, 7.2 Hz), 7.31 (td, 1 H, *J* = 7.2, 0.6 Hz), 6.98 (ddd, 1 H, *J* = 9, 2.4, 1.8 Hz), 5.33 (s, 2 H), 3.45–3.42 (m, 2 H), 3.11–3.07 (m, 2 H); ^13^C-NMR (CD_3_OD, 150 MHz) δ 164.7, 155.7, 153.7, 150.7, 146.9, 139.0, 129.5 (2 C), 129.5 (2 C), 129.1, 126.8, 120.7, 118.3, 112.9, 110.7, 78.1, 38.7, 31.2.

#### 2,3,4,9-Tetrahydro-6-hydroxy-1H-pyrido[3,4-b]indol-1-one (**kb-NB123-59**)

[63]. Yield: 95%; m.p. 244–247 °C; IR (ATR, neat) 3391, 3267, 1650, 1618, 1498, 1335, 1207 cm^−1; 1^H-NMR (DMSO-*d_6_*, 600 MHz) δ 11.26 (s, 1 H), 8.85 (d, 1 H, *J* = 2.4 Hz), 7.47 (s, 1 H), 7.18 (dd, 1 H, *J* = 8.4, 3.0 Hz), 6.83 (d, 1 H, *J* = 2.4 Hz), 6.74 (dd, 1 H, *J* = 8.4, 2.4 Hz), 3.50–3.43 (m, 2 H), 2.84–2.79 (m, 2 H); ^13^C-NMR (DMSO-*d_6_*, 150 MHz) δ 162.0, 150.9, 131.8, 127.5, 125.5, 117.1, 115.1, 113.0, 103.0, 41.2, 20.4; HRMS (EI) *m/z* calcd for C_11_H_10_N_2_O_2_ (M^+^) 202.0742, found 202.0752.

#### 3,4,5,10-Tetrahydro-7-hydroxy-azepino[3,4-b]indol-1(2H)-one (**kb-NB123-57**)

Yield: 89%; IR (ATR, neat) 3362, 3276, 1600, 1545, 1484, 1362 cm^−1; 1^H-NMR (DMSO-*d*_6_, 300 MHz) δ 10.82 (s, 1 H), 8.78 (s, 1 H), 7.86 (t, 1 H, *J* = 4.5 Hz), 7.19 (d, 1 H, *J* = 8.7 Hz), 6.79 (d, 1 H, *J* = 1.8 Hz), 6.73 (dd, 1 H, *J* = 8.7, 2.3 Hz), 3.30–3.22 (m, 2 H), 2.91 (t, 2 H, *J* = 6.3 Hz), 2.07–1.94 (m, 2 H); ^13^C-NMR (DMSO-*d*_6_, 150 MHz) δ 164.2, 150.6, 130.5, 128.1, 127.6, 115.7, 115.2, 112.7, 102.8, 41.6, 26.9, 25.4; HRMS (EI) *m/z* calcd for C_12_H_12_N_2_O_2_ (M^+^) 216.0899, found 216.0898.

#### 2,3,4,9-Tetrahydro-6-benzyloxy-1H-pyrido[3,4-b]indol-1-one (**kb-NB123-52**)

[63]. Yield: 52%; m.p. 208–210 °C; IR (ATR, neat) 3233, 2905, 2691, 1567, 1508, 1379, 1242 cm^−1; 1^H-NMR (DMSO-*d_6_*, 600 MHz) δ 11.45 (s, 1 H), 7.53 (s, 1 H), 7.47 (d, 2 H, *J* = 7.2 Hz), 7.39 (t, 2 H, *J* = 7.8 Hz), 7.32 (t, 1 H, *J* = 7.2 Hz), 7.29 (d, 1 H, *J* = 9.0 Hz), 7.17 (d, 1 H, *J* = 1.8 Hz), 6.94 (dd, 1 H, *J* = 9.0, 1.8 Hz),5.10 (s, 2 H), 3.48 (td, 2 H, *J* = 6.6, 1.8 Hz), 2.87 (t, 2 H, *J* = 6.6 Hz); ^13^C-NMR (DMSO-*d_6_*, 150 MHz)δ 161.9, 152.6, 137.6, 132.5, 128.4 (2 C), 127.8, 127.7 (2 C), 127.7, 125.1, 117.7, 115.6, 113.4, 102.4,69.7, 41.2, 20.5; HRMS (EI) *m/z* calcd for C_18_H_16_N_2_O_2_ (M^+^) 292.1212, found 292.1223.

#### 3,4,5,10-Tetrahydro-7-benzyloxy-azepino[3,4-b]indol-1(2H)-one (**kb-NB123-53**)

Yield: 53%; representative experimental data: IR (ATR, neat) 3227, 3194, 3033, 2920, 1623, 1543, 1478, 1453, 1276, 1197 cm^−1; 1^H-NMR (DMSO-*d*_6_, 600 MHz) δ 11.03 (s, 1 H), 7.95 (bs, 1 H), 7.50–7.42 (d, 2 H), 7.41–7.33 (m, 2 H), 7.32–7.29 (m, 2 H), 7.11 (bs, 1 H), 6.97–6.91 (m, 1 H), 5.10 (s, 2 H), 3.38–3.34 (m, 2 H), 2.96 (bs, 2 H), 2.02 (bs, 2 H); ^13^C-NMR (DMSO-*d*_6_, 150 MHz) δ 164.1, 152.3, 137.6, 131.3, 128.4, 127.8, 127.7, 127.6, 116.4, 115.6, 113.0, 102.2, 69.7, 41.6, 26.8, 25.4; HRMS (EI) *m/z* calcd for C_19_H_18_N_2_O_2_ (M^+^) 306.1368, found 306.1366.

#### 6-Amino-2,3,4,9-tetrahydro-1H-pyrido[3,4-b]indol-1-one (**kb-NB142-08**)

Yield: 69%; m.p. 280–282 °C (lit. 280–282 °C) [65]; IR (ATR, neat) 3356, 3230, 1653, 1501, 1327, 1225 cm^−1; 1^H-NMR (CD_3_OD, 600 MHz) δ 7.25 (d, 1 H, *J* = 8.4 Hz), 6.93 (s, 1 H), 6.86 (d, 1 H, *J* = 8.4 Hz), 3.62 (t, 2 H, *J* = 7.2 Hz), 2.94 (t, 2 H, *J* = 7.2 Hz); ^13^C-NMR (CD_3_OD, 150 MHz) δ 165.2, 141.1, 134.4, 127.6, 127.2, 120.0, 118.5, 113.9, 105.9, 42.8, 21.6; HRMS (EI) *m/z* calcd for C_11_H_11_N_3_O (M^+^) 201.0902,found 201.0901.

#### 6-Amino-3,4,5,10-tetrahydro-1H-azepino[3,4-b]indol-1(2H)-one (**kb-NB142-01**)

Yield: 68% m.p. 202–207 °C; IR (ATR, neat) 3208, 2921, 1618, 1542, 1479, 1450, 1335, 1296 cm^−1; 1^H-NMR (DMSO-*d_6_*, 600 MHz) δ 10.85 (s, 1 H), 7.93–7.85 (m, 1 H), 7.22–7.17 (m, 1 H), 6.86–6.83 (m, 1 H), 6.78–6.74 (m, 1 H), 6.39 (bs, 2 H), 3.30–3.24 (m, 2 H), 2.94–2.88 (m, 2 H), 2.04–1.98 (m, 2 H); ^13^C-NMR (DMSO-*d_6_*, 150 MHz) δ 164.3, 140.7, 130.0, 128.3, 127.0, 115.8, 115.1, 112.4, 101.9, 41.6, 27.0, 25.5; HRMS (EI) *m/z* calcd for C_12_H_13_N_3_O (M^+^) 215.1059, found 215.1062.

#### N-(2,3,4,9-Tetrahydro-1-oxo-1H-pyrido[3,4-b]indol-6-yl)-acetamide (**kb-NB123-93**)

Yield: 24%; m.p. >300 °C (lit > 320 °C) [65]; IR (ATR, neat) 3206, 1642, 1588, 1542, 1484, 1437, 1270 cm^−1; 1^H-NMR (DMSO-*d_6_*, 600 MHz) δ 11.50 (s, 1 H), 9.80 (s, 1 H), 7.91 (s, 1 H), 7.54 (s, 1 H), 7.32–7.22 (m, 2 H), 3.52–3.45 (m, 2 H), 2.89–2.83 (m, 2 H), 2.03 (s, 3 H); ^13^C-NMR (DMSO-*d_6_*, 150 MHz) δ 167.8, 161.8, 133.8, 131.9, 127.8, 124.6, 118.0, 117.9, 112.4, 109.8, 41.1, 23.9, 20.4; HRMS (EI) *m/z* calcd for C_13_H_13_N_3_O_2_ (M^+^) 243.1008, found 243.1009.

#### N-(3,4,5,10)-Tetrahydro-1-oxo-1H-azepino[3,4-b]indol-7-yl)-(2H)-acetamide (**kb-NB123-94**)

Yield: 75%; m.p. > 300 °C; IR (ATR, neat) 3270, 1618, 1547, 1477, 1453, 1267 cm^−1; 1^H-NMR (DMSO-*d_6_*, 600 MHz) δ 11.06 (s, 1 H), 9.80 (s, 1 H), 7.95 (t, 1 H, *J* = 4.5 Hz), 7.88 (s, 1 H), 7.32–7.25 (m, 2 H), 3.30–3.26 (m, 2 H), 2.95 (t, *J* = 6.6 Hz, 2 H), 2.00–2.06 (m, 5 H); ^13^C-NMR (DMSO-*d_6_*, 150 MHz) δ 167.7, 164.1, 132.6, 131.5, 127.8, 127.2, 118.1, 116.6, 112.0, 109.7, 41.5, 26.9, 25.4, 23.9; HRMS (EI) *m/z* calcd for C_14_H_15_N_3_O_2_ (M^+^) 257.1164, found 257.1166.

#### 3,4-Dihydro-9-benzyloxy-[1]benzothieno[2,3-f]-1,4-thiazepin-5(2H)-one (**kb-NB123-66**)

Yield: 87%; representative experimental data: m.p. 247–249 °C; IR (ATR, neat) 3165, 3037, 1650, 1500, 1282 cm^−1; 1^H-NMR (DMSO-*d*_6_, 600 MHz) δ 8.47 (t, 1 H, *J* = 5.6 Hz), 7.90 (d, 1 H, *J* = 8.8 Hz), 7.49 (d, 2 H, *J* = 7.5 Hz), 7.40 (t, 2 H, *J* = 7.7 Hz), 7.36–7.32 (m, 1 H), 7.29–7.27 (m, 1 H), 7.26–7.23 (m, 1 H), 5.20 (s, 2 H), 3.64–3.60 (m, 2 H), 3.41–3.37 (m, 2 H); ^13^C-NMR (DMSO-*d*_6_, 150 MHz) δ 165.1, 156.4, 139.4, 136.9, 133.3, 131.2, 128.5, 127.9, 127.8, 123.8, 118.0, 105.8, 69.6, 42.4, 33.4; HRMS (EI) *m/z* calcd for C_18_H_15_NO_2_S_2_ (M^+^) 341.0544, found 341.0543.

#### 3,4-Dihydro-9-hydroxy-[1]benzothieno[2,3-f]-1,4-thiazepin-5(2H)-one (**kb-NB142-70**)

Yield: 93%; representative experimental data: m.p. 218–220 °C (dec., dark brown), 235–238 °C (dec.); IR (ATR, neat) 3269, 1633, 1597, 1496, 1432, 1197 cm^−1; 1^H-NMR (DMSO-*d*_6_, 600 MHz) δ 9.73 (s, 1 H), 8.42 (t, 1 H, *J* = 5.5 Hz), 7.77 (d, 1 H, *J* = 8.7 Hz), 7.11 (d, 1 H, *J* = 1.9 Hz), 7.01 (dd, 1 H, *J* = 8.7, 1.6 Hz), 3.64–3.59 (m, 2 H), 3.40–3.36 (m, 2 H); ^13^C-NMR (DMSO-*d*_6_, 150 MHz) δ 165.2, 155.3, 139.6, 132.8, 129.3, 127.l, 123.6, 117.8, 107.0, 42.5, 33.3; HRMS (EI) *m/z* calcd for C_11_H_9_NO_2_S_2_ (M^+^) 251.0075,found 251.0080.

#### 3,4-Dihydro-9-methoxy-[1]benzothieno[2,3-f]-1,4-thiazepin-5(2H)-one (**kb-NB165-09**)

[68]. Yield: 92%; m.p. 202–204 °C (lit. 209–209.5 °C) [78]; IR (ATR, neat) 3156, 3018, 2916, 1633, 1499, 1403, 1284 cm^−1; 1^H-NMR (DMSO-*d*_6_, 600 MHz) δ 8.47 (t, 1 H, *J* = 5.3 Hz), 7.89 (d, 1 H, *J* = 8.3 Hz), 7.19–7.15 (m, 2 H), 3.84 (s, 3 H), 3.65–3.60 (m, 2 H), 3.42–3.38 (m, 2 H); ^13^C-NMR (DMSO-*d*_6_, 150 MHz) δ 165.2, 157.4, 139.4, 133.4, 130.9, 127.7, 123.8, 117.5, 104.4, 55.4, 42.4, 33.4; HRMS (EI) *m/z* calcd for C_12_H_11_NO_2_S_2_ (M^+^) 265.0231, found 265.0232.

#### 3,4-Dihydro-9-amino-[1]benzothieno[2,3-f]-1,4-thiazepin-5(2H)-one (**mcf292-03**)

Yield: 71%; m.p. 198.6–199.0 °C; IR (ATR, neat) 3370, 3254, 3146, 3008, 2915, 1623, 1599, 1556, 1491, 1454, 1430, 1403, 1346, 1333, 1312, 1286, 1243, 1204, 1184, 1129, 1083, 975, 887, 837, 798, 766, 749, 719, 691, 677, 663 cm^−1; 1^H-NMR (CD_3_OD, 400 MHz) δ 7.58 (dd, 1 H, *J* = 8.8, 0.4 Hz), 7.15 (dd, 1 H, *J* = 2.4, 0.4 Hz), 6.98 (dd, 1 H, *J* = 8.8, 2.0 Hz), 3.77–3.71 (m, 2 H), 3.42–3.37 (m, 2 H); ^13^C-NMR (CD_3_OD, 100 MHz) δ 169.3, 147.0, 141.5, 132.4, 130.8, 130.7, 124.0, 119.7, 108.4, 44.3, 35.1; MS (EI) *m/z* 250 (M^+^, 100); HRMS (EI) *m/z* calcd for C_11_H_10_N_2_OS_2_ (M^+^) 250.0235, found 250.0225.

#### 3,4-Dihydro-9-azido-[1]benzothieno[2,3-f]-1,4-thiazepin-5(2H)-one (**mcf292-08**)

Yield: 53%, 96% purity estimated by ^1^H-NMR; m.p. 193 °C (dec.); IR (ATR, neat) 3260, 3154, 3016, 2922, 2115, 1631, 1592, 1495, 1467, 1441, 1422, 1400, 1340, 1284, 1252, 1234, 1215, 1198, 1144, 1113, 975, 889, 835, 809, 792, 751, 721 cm^−1; 1^H-NMR (DMSO-*d*_6_, 400 MHz) δ 8.53 (bt, 1 H, *J* = 5.6 Hz), 8.06 (d, 1 H, *J* = 8.8 Hz), 7.39 (d, 1 H, *J* = 1.6 Hz), 7.31 (dd, 1 H, *J* = 8.6, 2.2 Hz), 3.68–3.60 (m, 2 H), 3.45–3.38 (m, 2 H); ^13^C-NMR (DMSO-*d*_6_, 100 MHz) δ 164.9, 139.4, 136.9, 135.2, 134.2, 127.7, 124.6, 119.2, 112.0, 42.4, 33.4; MS (EI) *m/z* 276 (M^+^, 14), 248 ([M–N_2_]^+^, 50), 68 (100); HRMS (EI) *m/z* calcd for C_11_H_8_N_4_OS_2_ (M^+^) 276.0140, found 276.0137.

#### 3,4-Dihydro-9-isothiocyanato-[1]benzothieno[2,3-f]-1,4-thiazepin-5(2H)-one (**mcf292-05**)

Yield: 58%, 98% purity estimated by ^1^H-NMR; m.p. 253.0–253.3 °C (softening point: 251.6 °C); IR (ATR, neat) 3260, 3155, 3025, 2922, 2067 (broad), 1633, 1590, 1497, 1469, 1457, 1441, 1420, 1400, 1340, 1321, 1282, 1254, 1241, 1141, 941, 900, 852, 807, 792, 744 cm^−1; 1^H-NMR (DMSO-*d*_6_, 400 MHz) δ 8.56 (bt, 1 H, *J* = 5.8 Hz), 8.11 (dd, 1 H, *J* = 8.8, 0.4 Hz), 7.80 (dd, 1 H, *J* = 2.0, 0.4 Hz), 7.58 (dd, 1 H, *J* = 8.8, 2.0 Hz), 3.68–3.61 (m, 2 H), 3.46–3.39 (m, 2 H); ^13^C-NMR (DMSO-*d*_6_, 100 MHz) δ 164.7, 139.0, 137.7, 134.4, 128.2, 127.2, 124.9, 124.5, 119.7, 42.4, 33.3; MS (EI) *m/z* 292 (M^+^, 47), 113 (57), 95 (86), 83 (96), 81 (100); HRMS (EI) *m/z* calcd for C_12_H_8_N_2_OS_3_ (M^+^) 291.9799, found 291.9795.

#### 3,4-Dihydro-9-(2-chloroacetamido)-[1]benzothieno[2,3-f]-1,4-thiazepin-5(2H)-one (**mcf292-09**)

Yield: 65%; m.p. 229–230 °C (dec.); IR (ATR, neat) 3381, 3262, 3155, 3010, 2928, 1668, 1649, 1635, 1571, 1523, 1495, 1467, 1446, 1403, 1338, 1277, 1266, 1243, 1187, 1144, 986, 891, 876, 816, 790, 773, 734, 729 cm^−1; 1^H-NMR (DMSO-*d*_6_, 300 MHz) δ 8.48 (bt, 1 H, *J* = 5.8 Hz), 8.25 (d, 1 H, *J* = 1.5 Hz), 7.95 (d, 1 H, *J* = 8.7 Hz), 7.63 (dd, 1 H, *J* = 8.7, 2.1 Hz), 4.29 (s, 2 H), 3.70–3.58 (m, 2 H), 3.45–3.38 (m, 2 H); ^13^C-NMR (DMSO-*d*_6_, 100 MHz) δ 165.0, 164.8, 138.7, 135.9, 133.7, 132.9, 128.2, 123.2, 119.8, 112.4, 43.6, 42.5, 33.2; MS (EI) *m/z* 328 (40), 326 (M^+^, 100); HRMS (EI) *m/z* calcd for C_13_H_11_ClN_2_O_2_S_2_ (M^+^) 325.9950, found 325.9952.

#### 3,4-Dihydro-9-hydroxy-10-iodo-[1]benzothieno[2,3-f]-1,4-thiazepin-5(2H)-one (**kb-NB165-31**)

Yield: 66%; m.p. 128 °C (dec.); IR (ATR, neat) 3335, 3071 (br), 1595, 1485, 1387, 1292 cm^−1; 1^H-NMR (DMSO-*d_6_*, 600 MHz) δ 10.52 (s, 1 H), 8.54 (t, 1 H, *J* = 5.4 Hz), 7.80 (d, 1 H, *J* = 8.4 Hz), 7.11 (d, 1 H, *J* = 8.4 Hz), 3.47 (q, 2 H, *J* = 5.4 Hz), 3.30–3.26 (m, 2 H); ^13^C-NMR (DMSO-*d_6_*, 150 MHz) δ 165.1, 155.2, 138.6, 138.3, 132.0, 128.7, 123.9, 115.4, 78.7, 42.2, 35.5; HRMS (EI) *m/z* calcd for C_11_H_8_INO_2_S_2_ (M^+^) 376.9041, found 376.9045.

#### 3,4-Dihydro-9-hydroxy-10-bromo-[1]benzothieno[2,3-f]-1,4-thiazepin-5(2H)-one (**kb-NB184-52**)

Yield: 25%; m.p. 235–238 °C (dec., bprown); IR (ATR, neat) 3350, 3083, 2918, 1595 1492, 1396, 1303 cm^−1; 1^H-NMR (DMSO-*d_6_*, 600 MHz) δ 10.38 (s, 1 H), 8.52 (t, 1 H, *J* = 5.4 Hz), 7.78 (d, 1 H, *J* = 8.4 Hz), 7.16 (d, 1 H, *J* = 8.4 Hz), 3.53 (q, 2 H, *J* = 5.4 Hz), 3.31–3.27 (m, 2 H); ^13^C-NMR (DMSO-*d_6_*, 150 MHz) δ 164, 152.6, 136.5, 136.4, 132.0, 129.1, 122.8, 117.1, 103.3, 42.6, 34.8; HRMS (EI) *m/z* calcd for C_11_H_8_BrNO_2_S_2_ (M^+^) 328.9149 found 328.9164.

#### 3,4-Dihydro-7-benzyloxy-[1]benzothieno[2,3-f]-1,4-thiazepin-5(2H)-one (**kb-NB184-38**)

Yield: 53%; m.p. 213–215 °C; IR (ATR, neat) 3159, 3036, 2923, 1647, 1462, 1257 cm^−1; 1^H-NMR (DMSO-*d_6_*, 600 MHz) δ 8.47 (t, 1 H, *J* = 5.4 Hz), 7.49 (d, 2 H, *J* = 7.2 Hz), 7.22–7.31 (m, 5 H), 7.19 (d, 1 H, *J* = 3.0 Hz), 5.35 (s, 2 H), 3.68–3.62 (m, 2 H), 3.42–3.38 (m, 2 H); ^13^C-NMR (DMSO-*d_6_*, 150 MHz) δ 165.0, 152.7, 140.0, 136.6, 132.1, 129.0, 128.6 (2 C), 128.0, 127.9, 127.4 (2 C), 126.6, 115.4, 108.7, 69.7, 42.6, 33.1; HRMS (EI) *m/z* calcd for C_18_H_15_NO_2_S_2_ (M^+^) 341.0544, found 341.0543.

#### 3,4-Dihydro-7-hydroxy-[1]benzothieno[2,3-f]-1,4-thiazepin-5(2H)-one (**kb-NB184-40**)

Yield: 73%; m.p. 269–272 °C (dec., dark brown), 278–281 °C (dec.); IR (ATR, neat) 3255, 3155, 3015, 1620, 1439, 1285 cm^−1; 1^H-NMR (DMSO-*d_6_*, 600 MHz) δ 10.55 (s, 1 H), 8.45 (t, 1 H, *J* = 5.4 Hz), 7.29 (t, 1 H, *J* = 7.8 Hz), 7.24 (d, 1 H, *J* = 7.9 Hz), 6.89 (d, 1 H, *J* = 7.5 Hz), 3.65–3.60 (m, 2 H), 3.40–3.36 (m, 2 H); ^13^C-NMR (DMSO-*d_6_*, 150 MHz) δ 165.2, 152.1, 140.3, 131.8, 128.8, 126.65, 126.61, 113.7, 110.8, 42.5, 33.2; HRMS (EI) *m/z* calcd for C_11_H_9_NO_2_S_2_ (M^+^) 251.0075, found 251.0066.

#### 3,4-Dihydro-7-methoxy-[1]benzothieno[2,3-f]-1,4-thiazepin-5(2H)-one (**kb-NB184-44**)

Yield: 99%; m.p. 220–224 °C; IR (ATR, neat) 3163, 3032, 2932, 1634, 1467, 1261 cm^−1; 1^H-NMR (DMSO-*d_6_*, 600 MHz) δ 8.48 (t, 1 H, *J* = 4.8 Hz), 7.44 (t, 1 H, *J* = 7.8 Hz), 7.37 (d, 1 H, *J* = 7.8 Hz), 7.09 (d, 1 H, *J* = 7.8 Hz), 3.96 (s, 3 H), 3.66–3.61 (m, 2 H), 3.42–3.37 (m, 2 H); ^13^C-NMR (DMSO-*d_6_*, 150 MHz) δ 165.0, 153.8, 139.9, 132.0, 128.9, 127.4, 126.7, 115.1, 107.2, 55.9, 42.5, 33.1; HRMS (ESI) *m/z* calcd for C_12_H_11_NO_2_S_2_ ([M+Na]^+^) 288.0129, found 288.0102.

#### 3,4-Dihydro-9-hydroxy-6-oxide-[1]benzothieno[2,3-f]-1,4-thiazepin-5(2H)-one (**kb-NB184-22**)

Yield: 80%; m.p. 160–163 °C (dec., brown) 280–282 °C (dec., melts); IR (ATR, neat) 3369 (br), 1648, 1608, 1577, 1452, 1335, 1245, 998 cm^−1; 1^H-NMR (DMSO-*d_6_*, 600 MHz) δ 9.90 (bs, 1 H), 7.80 (bs, 1 H), 7.47 (s, 1 H), 7.33 (d, 1 H, *J* = 9.0 Hz), 7.04 (d, 1 H, *J* = 9.0 Hz), 3.73–3.63 (m, 2 H), 3.00–2.90 (m, 2 H); ^13^C-NMR (DMSO-*d_6_*, 150 MHz) δ 175.8, 156.2, 146.6, 127.3, 123.5, 121.7, 118.1, 109.5, 101.7, 42.6, 22.7; MS (ESI) *m/z* 268 ([M+H]^+^).

#### 3,4-Dihydro-9-methoxy-6-oxide-[1]benzothieno[2,3-f]-1,4-thiazepin-5(2H)-one (**kb-NB184-25**)

Yield: 58%; m.p. 284–287 °C; IR (ATR, neat) 3340, 1506, 1477, 1337, 1248 cm^−1; 1^H-NMR (DMSO-*d_6_*, 600 MHz) δ 7.93 (bs, 1 H), 7.60 (d, 1 H, *J* = 2.4 Hz), 7.46 (d, 1 H, *J* = 9.0 Hz), 7.18 (dd, 1 H, *J* = 9.0, 2.4 Hz), 3.86 (s, 3 H), 3.75–3.69 (m, 2 H), 3.00–2.95 (m, 2 H); ^13^C-NMR (DMSO-*d_6_*, 150 MHz) δ 175.7, 158.1, 146.5, 127.5, 123.7, 123.2, 117.3, 107.4, 102.0, 55.8, 42.6, 22.7; MS (ESI) *m/z* 282 ([M+H]^+^); HRMS (EI) *m/z* calcd for C_12_H_11_NO_3_S_2_ (M^+^) 281.0180, found 281.0180.

#### 3,4-Dihydro-9-methoxy-1-oxide-[1]benzothieno[2,3-f]-1,4-thiazepin-5(2H)-one (**kb-NB184-45**)

Yield: 54%; m.p. 215–217 °C; IR (ATR, neat) 3156, 3020, 2915, 1638, 1507 cm^−1; 1^H-NMR (DMSO-*d_6_*, 600 MHz) δ 8.99 (bs, 1 H), 8.03 (d, 1 H, *J* = 9.0 Hz), 7.59 (d, 1 H, *J* = 2.4 Hz), 7.25 (dd, 1 H, *J* = 8.4, 2.4 Hz), 3.94–3.87 (m, 1 H), 3.87 (s, 3 H), 3.59–3.51 (m, 3 H); ^13^C-NMR (DMSO-*d_6_*, 150 MHz) δ 162.4, 157.8, 141.9, 140.1, 133.6, 131.5, 124.0, 117.9, 104.6, 55.5, 50.4, 32.6; HRMS (EI) *m/z* calcd for C_12_H_11_NO_3_S_2_ (M^+^) 281.0180, found 281.0177.

#### 2,3,4,5-Tetrahydro-10-benzyloxybenzo[b]thieno[2,3-f]-1,5-thiazocin-6-one (**kb-NB165-89**)

Yield: 42%; m.p. 198–199 °C; IR (ATR, neat) 3162, 3033, 2937, 1644, 1619, 1600, 1497, 1384, 1274, 1193 cm^−1; 1^H-NMR (DMSO-*d*_6_, 600 MHz) δ 8.02 (bs, 1 H), 7.90–7.84 (m, 1 H), 7.51–7.45 (m, 2 H), 7.43–7.37 (m, 2 H), 7.36–7.31 (m, 1 H), 7.26–7.19 (m, 2 H), 5.20 (s, 2 H), 3.50–3.43 (m, 2 H), 3.30–3.24 (m, 2 H), 1.92–1.89 (m, 2 H); ^13^C-NMR (DMSO-*d*_6_, 150 MHz) δ 164.8, 156.4, 138.7, 136.9, 130.3, 128.5, 128.5, 127.9, 127.7, 127.4, 123.6, 117.3, 105.8, 69.6, 30.5, 27.4; MS (EI) *m/z* 356 (23), 355 (M^+^, 100), 357 (12); HRMS (EI) *m/z* calcd for C_19_H_17_NO_2_S_2_ (M^+^) 355.0701, found 355.0689.

#### 2,3,4,5-Tetrahydro-10-hydroxybenzo[b]thieno[2,3-f]-1,5-thiazocin-6-one (**kb-NB165-92**)

Yield: 92%; m.p. 139–142 °C; IR (ATR, neat) 3256 (br), 3169 (br), 1615, 1492, 1444, 1182 cm^−1; 1^H-NMR (DMSO-*d*_6_, 600 MHz) δ 9.71 (s, 1 H), 7.97 (t, 1 H, *J* = 7.8 Hz), 7.74 (d, 1 H, *J* = 8.6 Hz), 7.09 (s, 1 H), 6.98 (d, 1 H, *J* = 8.6 Hz), 3.50–3.43 (m, 2 H), 3.30–3.23 (m, 2 H), 1.92–1.85 (m, 2 H); ^13^C-NMR (DMSO-*d*_6_, 150 MHz) δ 164.9, 155.3, 139.0, 128.4, 127.0, 123.4, 117.1, 106.8, 30.5, 27.3; MS (EI) *m/z* 266 (15), 265 (M^+^, 100), 267 (11); HRMS (EI) *m/z* calcd for C_12_H_11_NO_2_S_2_ (M^+^) 265.0231, found 265.0230.

#### 2,3,4,5-Tetrahydro-10-methoxybenzo[b]thieno[2,3-f]-1,5-thiazocin-6-one (**kb-NB184-02**)

Yield: 77%; m.p. 185–188 °C; IR (ATR, neat) 3152, 3026, 2939, 1636, 1498, 1395, 1209 cm^−1; 1^H-NMR (DMSO-*d*_6_, 600 MHz) δ 8.02 (t, 1 H, *J* = 7.1 Hz), 7.87 (d, 1 H, *J* = 9.4 Hz), 7.16–7.13 (m, 2 H), 3.84 (s, 3 H), 3.50–3.44 (m, 2 H), 3.30–3.26 (m, 2 H), 1.92–1.87 (m, 2 H); ^13^C-NMR (DMSO-*d*_6_, 150 MHz) δ 164.8, 157.4, 138.7, 130.1, 127.4, 123.6, 116.8, 104.3, 55.4, 30.5, 27.4; MS (EI) *m/z* 280 (16), 279 (M^+^, 100); HRMS (EI) *m/z* calcd for C_13_H_13_NO_2_S_2_ (M^+^) 279.0388, found 279.0379.

#### 2,3,4,5-Tetrahydro-10-benzyloxybenzo[b]thieno[2,3-f]-1,5-oxazocin-6-one (**kb-NB184-36**)

Yield: 51%; m.p. 195–200 °C; IR (ATR, neat) 2912 (br), 2298, 1637, 1607, 1528, 1455, 1422 1222 cm^−1; 1^H-NMR (DMSO-*d_6_*, 600 MHz) δ 8.00 (t, 1 H, *J* = 7.2 Hz), 7.75 (d, 1 H, *J* = 8.4 Hz), 7.47 (d, 2 H, *J* = 7.8 Hz), 7.40 (t, 2 H, *J* = 7.8 Hz), 7.37–7.32 (m, 1 H), 7.26 (d, 1 H, *J* = 2.4 Hz), 7.18 (dd, 1 H, *J* = 8.4, 2.4 Hz), 5.17 (s, 2 H), 4.49 (t, 2 H, *J* = 5.4 Hz), 3.40–3.35 (m, 2 H), 1.87 (quint, 2 H, *J* = 5.4 Hz); ^13^C-NMR (DMSO-*d_6_*, 150 MHz) δ 165.1, 156.2, 149.2, 137.0, 133.5, 129.2, 128.5 (2 C), 127.9, 127.6 (2 C), 123.8, 118.3, 112.0, 104.7, 69.5, 68.6, 37.8, 29.2; HRMS (ESI) *m/z* calcd for C_19_H_17_NO_3_S ([M+Na]^+^) 362.0827, found 362.0809.

#### 2,3,4,5-Tetrahydro-10-methoxybenzo[b]thieno[2,3-f]-1,5-oxazocin-6-one (**kb-NB184-57**)

Yield: 35%; m.p. 233–237 °C; IR (ATR, neat) 3154, 3027, 2919, 1631, 1467, 1422, 1220 cm^−1; 1^H-NMR (DMSO-*d_6_*, 600 MHz) δ 8.00 (t, 1 H, *J* = 6.6 Hz), 7.73 (d, 1 H, *J* = 9.0 Hz), 7.15 (d, 1 H, *J* = 2.4 Hz), 7.09 (dd, 1 H, *J* = 9.0, 2.4 Hz), 4.50 (t, 2 H, *J* = 5.4 Hz), 3.81 (s, 3 H), 3.42–3.32 (m, 2 H), 1.92–1.84 (m, 2 H); ^13^C-NMR (DMSO-*d_6_*, 150 MHz) δ 165.1, 157.2, 149.2, 133.5, 128.9, 123.7, 117.9, 112.0, 103.3, 68.6, 55.3, 37.8, 29.2; HRMS (ESI) *m/z* calcd for C_13_H_13_NO_3_S ([M+Na]^+^) 286.0514, found 286.0510.

#### Methyl 3-(3-hydroxypropylamino)-5-methoxybenzo[b]thiophene-2-carboxylate (**kb-NB184-80**)

Yield: 71%; m.p. 116–120 °C; IR (ATR, neat) 3481, 2921, 1627, 1578, 1440, 1224 cm^−1; 1^H-NMR (CDCl_3_, 600 MHz) δ 7.60 (d, 1 H, *J* = 8.4 Hz), 7.57 (d, 1 H, *J* = 2.4 Hz), 7.11 (dd, 1 H, *J* = 8.4, 2.4 Hz), 3.87 (s, 3 H), 3.87 (s, 3 H), 3.90–3.87 (m, 2 H), 3.84 (t, 1 H, *J* = 6.6 Hz), 1.99 (quint, 2 H, *J* = 6.6 Hz), 1.25 (s, 2 H); ^13^C-NMR (CDCl_3_, 150 MHz) δ 166.4, 157.0, 151.8, 133.3, 133.0, 124.4, 118.1, 107.5, 101.9, 60.5, 55.8, 51.7, 43.7, 33.6; HRMS (EI) *m/z* calcd for C_14_H_17_NO_4_S (M^+^) 295.0878, found 295.0879.

#### 2,3-Dihydro-4-methyl-9-methoxy-[1]benzothieno[2,3-f]-1,4-thiazepin-5(2H)-one (**kb-NB165-16**)

Yield: 71%; m.p. 160–161 °C; IR (ATR, neat) 2928, 1625, 1598, 1497, 1397, 1207 cm^−1; 1^H-NMR (CD_3_OD, 600 MHz) δ 7.77 (d, 1 H, *J* = 9.0 Hz), 7.33 (d, 1 H, *J* = 2.4 Hz), 7.14 (dd, 1 H, *J* = 9.0, 2.4 Hz), 3.88 (s, 3 H), 3.86–3.83 (m, 2 H), 3.53–3.50 (m, 2 H), 3.22 (s, 3 H); ^13^C-NMR (CD_3_OD, 150 MHz) δ 167.5, 159.6, 141.3, 136.0, 133.2, 129.2, 124.5, 118.9, 105.7, 56.0, 51.3, 35.8, 35.0; HRMS (EI) *m/z* calcd for C_13_H_13_NO_2_S_2_ (M^+^) 279.0388, found 279.0386.

#### 2,3-Dihydro-4-methyl-9-hydroxy-[1]benzothieno[2,3-f]-1,4-thiazepin-5(2H)-one (**kb-NB165-17**)

Yield: 56%; m.p. 265–268 °C; IR (ATR, neat) 3193 (br), 2384, 1610, 1587, 1494, 1401 cm^−1; 1^H-NMR (CD_3_OD, 600 MHz) δ 7.69 (d, 1 H, *J* = 9.0 Hz), 7.26 (d, 1 H, *J* = 2.4 Hz), 7.03 (dd, 1 H, *J* = 9.0, 2.4 Hz), 3.85 (t, 2 H, *J* = 5.4 Hz), 3.50 (t, 2 H, *J* = 5.4 Hz), 3.21 (s, 3 H); ^13^C-NMR (CD_3_OD, 150 MHz) δ 167.6, 156.9, 141.5, 135.5, 131.9, 128.9, 124.4, 118.8, 108.5, 51.4, 35.8, 34.9; HRMS (EI) *m/z* calcd for C_12_H_11_NO_2_S_2_ (M^+^) 265.0231, found 265.0235.

#### 2,3-Dihydro-4-(2-aminoethyl)-9-hydroxy-[1]benzothieno[2,3-f]-1,4-thiazepin-5(2H)-one (**kb-NB165-75**)

Yield: quant.; m.p. 250–254 °C; IR (ATR, neat) 3251 (br), 2918 (br), 1579, 1500, 1427, 1178 cm^−1; 1^H-NMR (DMSO-*d_6_*, 600 MHz) δ 9.82 (s, 1 H), 7.99 (bs, 2 H), 7.79 (d, 1 H, *J* = 9.0 Hz), 7.18 (d, 1 H, *J* = 2.4 Hz), 7.04 (dd, 1 H, *J* = 9.0, 2.4 Hz), 3.85 (t, 2 H, *J* = 4.8 Hz), 3.73 (t, 2 H, *J* = 6.0 Hz), 3.48 (t, 2 H, *J* = 4.8 Hz), 3.10–3.03 (m, 2 H); ^13^C-NMR (DMSO-*d_6_*, 150 MHz) δ 165.2, 155.5, 139.5, 133.6, 129.3, 127.0, 123.7, 117.9, 107.1, 48.3, 45.8, 37.3, 32.8; HRMS (EI) *m/z* calcd for C_13_H_14_N_2_O_2_S_2_ (M^+^) 294.0497, found 294.0492.

#### 2,3,4,5-Tetrahydro-9-benzyloxy-[1]benzothieno[2,3-f]-1,4-thiazepine (**kb-NB165-81**)

Yield: 15%; m.p. 144–145 °C; IR (ATR, neat) 2915, 1596, 1443, 1270, 1192 cm^−1; 1^H-NMR (CD_3_OD, 600 MHz) δ 7.67 (d, *J* = 9.0 Hz, 1 H), 7.48 (d, 2 H, *J* = 7.2 Hz), 7.38 (t, 2 H, *J* = 7.2 Hz), 7.37–7.30 (m, 2 H), 7.06 (dd, 1 H, *J* = 9.0, 2.4 Hz), 5.14 (s, 2 H), 4.14 (s, 2 H), 3.44–3.41 (m, 2 H), 2.82–2.79 (m, 2 H); ^13^C-NMR (CD_3_OD, 150 MHz) δ 158.4, 145.0, 142.7, 138.7, 131.3, 129.5 (2 C), 128.9, 128.7 (2 C), 128.0, 124.2, 116.5, 107.2, 71.3, 55.3, 35.5; MS (EI) *m/z* 327 (M^+^); HRMS (EI) *m/z* calcd for C_18_H_17_NOS_2_ (M^+^) 327.0752, found 327.0749.

#### 2,3,4,5-Tetrahydro-9-hydroxy-[1]benzothieno[2,3-f]-1,4-thiazepine (**kb-NB165-83**)

Yield: 70%; m.p. 182–184 °C (dec., dark brown); IR (ATR, neat) 2947 (br), 2920, 1598, 1436, 1183 cm^−1; 1^H-NMR (CD_3_OD, 600 MHz) δ 7.59 (d, 1 H, *J* = 9.0 Hz), 7.16 (d, 1 H, *J* = 2.4 Hz,), 6.88 (dd, 1 H, *J* = 9.0, 2.4 Hz), 4.18 (s, 2 H), 3.49–3.45 (m, 2 H), 2.86–2.81 (m, 2 H); ^13^C-NMR (CD_3_OD, 150 MHz) δ 156.5, 143.9, 142.8, 130.1, 127.9, 124.1, 116.1, 108.4, 55.2, 35.1; MS (EI) *m/z* 238 (14), 237 (M^+^, 100), 239 (10); HRMS (EI) *m/z* calcd for C_11_H_11_NOS_2_ (M^+^) 237.0282, found 237.0289.

#### 2-Methoxy-7H,8H,9H-1,4-thiazepino [7′,6′-5,4]thiopheno[3,2-d]pyrimidin-6-one (**kmg-NB4-23**)

Yield: 68%; m.p. 308 °C (dec.); IR (ATR, neat) 3260, 3153, 3015, 1636, 1554, 1495, 1467, 1374, 1269, 1353, 1323 cm^−1; 1^H-NMR (DMSO-*d*_6_, 300 MHz) δ 9.36 (s, 1 H), 8.70 (t, 1 H, *J* = 5.4 Hz), 3.98 (s, 1 H), 3.68 (app dd, 2 H, *J* = 6.0 Hz), 3.40–3.36 (m, 2 H); ^13^C-NMR (DMSO-*d*_6_, 75 MHz) δ 164.2, 163.1, 159.6, 155.9, 138.5, 129.7, 124.1, 54.8, 42.7, 31.9; HRMS (ESI) *m/z* calcd for C_10_H_10_N_3_O_2_S_2_ ([M+H]^+^) 268.0214, found 268.0237.

#### 2-Hydroxy-7H,8H,9H-1,4-thiazepino[7′,6′-5,4]thiopheno[3,2-d]pyrimidin-6-one (hydrochloride salt, **kmg-NB4-69A**)

Yield: 85%; m.p. 335.9 °C (dec); IR (ATR) cm^−1^ 3452, 3267, 3176, 2591, 2032, 1912, 1700, 1623, 1463, 1240; ^1^H-NMR (DMSO-*d_6_*, 400 MHz) δ 9.20 (s, 1 H), 8.74 (t, 1 H, *J* = 6.2 Hz), 7.79 (bs, 1 H), 3.68–3.60 (m, 2 H), 3.39–3.32 (m, 2 H); ^13^C-NMR (DMSO-*d_6_*, 100 MHz) δ 164.0, 161.0, 159.7, 153.6, 140.9, 128.4, 119.6, 42.6, 32.2; HRMS (ESI) *m/z* calcd for C_9_H_8_N_3_O_2_S_2_ ([M+H]^+^) 254.0058, found 254.0041.

#### 2,4-Dimethoxy-7H,8H,9H-1,4-thiazepino[7′,6′-5,4]thiopheno[3,2-d]pyrimidin-6-one (**kmg-NB5-13**)

Yield: 77%; m.p. 288.0 °C (dec); IR (ATR, neat) 3321, 1642, 1579, 1545, 1491, 1476, 1458, 1346, 1331, 1206 cm^−1; 1^H-NMR (DMSO-*d_6_*, 500 MHz) δ 8.65 (t, 1 H, *J* = 5.7 Hz), 4.09 (s, 3 H), 3.96 (s, 3 H), 3.69–3.64 (m, 2 H), 3.38–3.34 (m, 2 H); ^13^C-NMR (DMSO-*d_6_*, 125 MHz) δ 165.5, 164.1, 163.5, 159.9, 135.9, 130.3, 111.5, 54.74, 54.72, 42.8, 31.8.

#### 4-Hydroxy-2-methoxy-7H,8H,9H-1,4-thiazepino[7′,6′-5,4]thiopheno[3,2-d]pyrimidin-6-one (**kmg-NB5-15**)

Yield: 77%; m.p. 295.0 °C (dec); IR (ATR, neat) 3266, 3170, 2740, 1674, 1646, 1603, 1465, 1407, 1316 cm^−1; 1^H-NMR (DMSO-*d_6_*, 400 MHz) δ 12.72 (s, 1 H), 8.56 (t, 1 H, *J* = 5.2 Hz), 3.94 (s, 3 H), 3.66–3.60 (m, 2 H), 3.32–3.28 (m, 2 H); ^13^C-NMR (DMSO-*d_6_*, 125 MHz) δ 164.0, 158.4, 156.8, 153.8, 135.7, 130.6, 118.6, 54.9, 42.7, 31.9; MS (EI) *m/z* 283 (M^+^, 100). HRMS (ESI) *m/z* calcd for C_10_H_9_N_3_O_3_S_2_Na ([M+Na]^+^) 305.9983, found 305.9995.

### 3.2. IMAP-based kinase counterscreening assay

An automated, HTS formatted IMAP-based PKD Fluorescence Polarization (FP) assay was used to assess the specificity of the PKD analogs as previously described [52]. Briefly, PKD1 kinase reactions were assembled by the addition of 3× concentrations of substrate/ATP (300 nm/60 μm), experimental compound, and PKD1 enzyme (0.18 milliunits/mL) in a miniaturized reaction volume (*i.e.*, 6 μL). Kinase reactions were incubated for 90 min at room temperature and stopped by the addition of IMAP binding reagent. Assay plates were then incubated for 2 h and IMAP-based FP data were captured on a Molecular Devices Spectra-Max M5 (Sunnyvale, CA, USA). The IC50 determinations for each compound in the PKD1 IMAP FP assay were conducted within the linear range of the captured signal readout (n = 3).

### 3.3. In vitro radiometric PKD kinase assays

*In vitro* radiometric PKD kinase assays were conducted as previously described [52]. Briefly, 50 ng recombinant human PKD1 (Biomol International, Plymouth Meeting, PA, USA) was incubated at 30 °C for 10 min with a reaction mixture containing 2.5 μg syntide-2 (Sigma), 70 μM ATP, and 1 μCi γ-^32^ P-ATP (PerkinElmer, Boston, MA, USA) in kinase buffer (50 mM Tris-HCl pH 7.5, 4 μM MgCl_2_, and 10 mM P-mercaptoethanol). 25 μL of the reaction mixture was then spotted on Whatman P81 filter paper (Whatman Inc., Clifton, NJ, USA), and filter papers were washed three times with 0.5% phosphoric acid, dried, then counted using a Beckman LS6500 multi-purpose scintillation counter.

### 3.4. Determination of cellular IC_50_ for PKD 1 inhibition

Inhibition of PKD 1 in cells was determined by densitometry analysis of Western blotting data for PKD1 autophosphorylation at Ser^916^ in LNCaP cells as previously described [52]. LNCaP cells were pre-treated with PKD inhibitors for 45 min at various concentrations, and then stimulated with 10 nM phorbol 12-myristate 13-acetate (PMA) for 20 min. Cells were then collected and lysed in lysis buffer containing 200 mM Tris-HCl, pH 7.4, 100 μM 4-(2-aminoethyl) benzenesulfonyl fluoride, 1 mM EGTA, and 1% Triton X-100. Cell lysates were probed by Western blot analysis using primary antibodies targeting p-S916-PKD1 (Millipore), PKD1 (Cell Signaling Technology), or GAPDH. Densitometry analysis of visualized bands was used to determine the cellular IC50 values for PKD1 inhibition.

### 3.5. Statistical analysis

GraphPad Prism V software was used to determine statistical significance. Each assay was repeated two or three times with triplicate determination at each point. A p value of < 0.05 was considered significant.

## 4. Conclusions

An extensive SAR study starting with the HTS-based confirmed hit **CID755673** led to the discovery of the benzothienothiazepinone **kb-NB142-70** and its methoxy analog **kb-NB165-09**, which were ca. 7-fold more potent at PKD1 inhibition. A 4-zone pharmacophore model was developed based on these new lead compounds, and systematic modifications of each zone led to a novel series of benzothienothiazepinones with equal or greater potencies compared to **CID755673**. Five of these analogs were further investigated, and each of them inhibited PMA-induced autophosphorylation of PKD1 [53]. These analogs also caused dramatic arrest in cell proliferation and inhibited cell migration and invasion [53]. Furthermore, counterscreens on a panel of other kinases clearly demonstrated a high selectivity profile in this series for PKD inhibition [53]. In order to decrease the susceptibility of these compounds to metabolic pathways, a pyrimidine moiety was installed in zone I, leading to a promising new lead structure, **kmg-NB4-23**. Because of the inadequate solubility properties of the pyrimidine-based inhibitors, additional modifications to zone I are currently being explored in our laboratories. Overall, the results of this extensive SAR study suggest that the zone I binding region is restrictive in both size and polarity of the aryl substituents. Additionally, it appears that the hydrogen bond donor-acceptor abilities of the amide moiety are critical for optimal interactions with the zone IV binding site. The synthesis of the azide analog **mcf292-08** and the electrophilic reactive probe molecules **mcf292-05** and **mcf292-09** as tools for photoaffinity/affinity labeling studies was undertaken to gain key structural insights into the possible protein binding site. The latter information is currently pending and will be utilized for future SAR studies.

## Figures and Tables

**Table 1.** Chemical structures and PKD1 inhibitory activities of **CID797718** and its analogs.

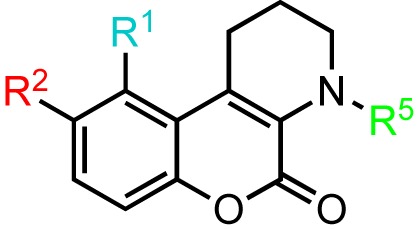						

**Entry**	**Compound**	**Structure**			**IC_50_**	
	
		**R^1^**	**R^2^**	**R^5^**	**IMAP-FP PKD1 (μM)**^a^	**Radiometric PKD1 (μM)**^b^
1	**CID797718**	H	OH	H	13.7 ± 0.42 (*n = 3*)	2.34 ± 0.16 (*n* = 3)
2	**kb-NB77-83**	H	OAllyl	H	not inhibitory	n.d.
3	**kb-NB77-78**	H	OTBS	H	not inhibitory	n.d.
4	**kb-NB77-91**	H	OH	Cbz	not inhibitory	n.d.
5	**kb-NB96-47-1**	Cl	OH	H	not inhibitory	n.d.

a PKD1 IC_50_ was determined using an automated, HTS formatted IMAP-based PKD Fluorescence Polarization (FP) assay as previously described [52]. Each IC_50_ was calculated as the mean ± SEM of at least three independent experiments with triplicate determinations at each concentration in each experiment; *n* = number of independent experiments.

b PKD1 IC_50_ was determined using a radiometric kinase activity assay as previously described [52]. Each IC_50_ was calculated as the mean ± SEM of at least three independent experiments with triplicate determinations at each concentration in each experiment; *n* = number of independent experiments.

**Table 2.** Chemical structure and PKD1 inhibitory activity of **CID755673** and its analogs.

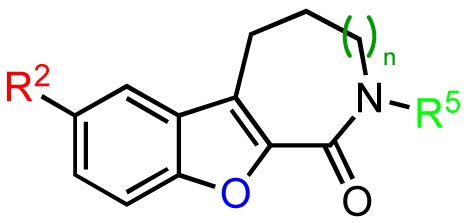							

**Entry**	**Compound**	**Structure**			**IC_50_**		

		**R^2^**	**R^5^**	**n**	**IMAP-FP PKD1 (μM)**^a^	**Radiometric PKD1 (μM)**^b^	**Cellular PKD1 (μM)**^c^
1	**CID755673**	OH	H	1	0.64 ± 0.03 (*n* = 3)	0.18 ± 0.02 (*n* = 5)	11.8 ± 4.0 (*n* = 3)
2	**kb-NB123-23A**	OH	H	0	12.6 ± 1.3 (*n* = 3)	1.41 (*n* = 1)	n.d.
3	**kb-NB123-32**	OMe	H	0	>100	n.d.	n.d.
4	**kb-NB96-53**	OH	H	2	8.3 ± 0.6 (*n* = 3)	1.03 (*n* = 1)	n.d.
5	**kb-NB96-59**	OMe	H	2	>100	n.d.	n.d.
6	**kb-NB77-56**	OMe	H	1	2.40 ± 0.14 (*n* = 3)	2.39 (*n* = 1)	n.d.
7	**kb-NB77-84**	OAllyl	H	1	2.6 ± 0.2 (*n* = 3)	1.23 (*n* = 1)	n.d.
8	**kb-NB123-36**	OAc	H	1	84.89 ± 3.21 (*n* = 3)	n.d.	n.d.
9	**kb-NB77-77**	OTBS	H	1	not inhibitory	n.d.	n.d.
10	**kb-NB123-37**	OMe	Me	0	not inhibitory	n.d.	n.d.
11	**kb-NB142-25**	OH	Me	1	n.d.	4.0 ± 1.1 (*n* = 2)	n.d.
12	**kb-NB96-04**	OMe	Me	1	>100	n.d.	n.d.
13	**kb-NB123-45-1**	OAc	Ac	1	not inhibitory	n.d.	n.d.
14	**kb-NB165-15**	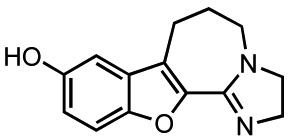			n.d.	not inhibitory	n.d.

a PKD1 IC_50_ was determined using an automated, HTS formatted IMAP-based PKD Fluorescence Polarization (FP) assay as previously described [52]. Each IC_50_ was calculated as the mean ± SEM of at least three independent experiments with triplicate determinations at each concentration in each experiment; *n* = number of independent experiments.

b PKD1 IC_50_ was determined using a radiometric kinase activity assay as previously described [52]. Each IC_50_ was calculated as the mean ± SEM with triplicate determinations at each concentration in each experiment; *n* = number of independent experiments.

c Cellular IC_50_ was determined by densitometry analysis of Western blotting data for PKD1 autophosphorylation at S^916^ in LNCaP cells as previously described [53]. Each IC_50_ was calculated as the mean ± SEM of at least two independent experiments; *n* = number of independent experiments.

**Table 3.** Chemical structures and PKD1 inhibitory activities of CID755673 analogs.

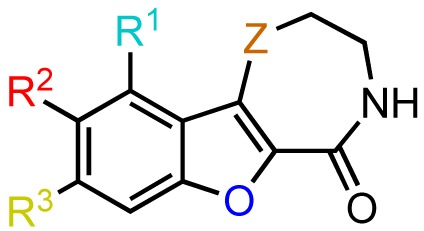							

**Entry**	**Compound**	**Structure**				**IC_50_**	
	
		**Z**	**R^1^**	**R^2^**	**R^3^**	**IMAP-FP PKD1 (*μ*M)**^a^	**Radiometric PKD1 (*μ*M)**^b^
1	**kb-NB77-88**	CH_2_	Cl	OH	H	1.4 ± 0.1 (*n* = 3)	0.89 (*n* = 1)
2	**kb-NB96-21**	CH_2_	F	OH	H	1.3 ± 0.05 (*n* = 3)	0.24 (*n* = 1)
3	**kb-NB96-50**	CH_2_	Cl	OAllyl	H	not inhibitory	n.d.
4	**kb-NB96-47-5**	CH_2_	H	OH	Cl	>100	n.d.
5	**kb-NB96-43**	CH_2_	Cl	OH	Cl	not inhibitory	n.d.
6	**kb-NB96-02**	CH_2_	Allyl	OH	H	2.4 ± 0.3 (*n* = 3)	1.58 (*n* = 1)
7	**kb-NB96-30**	CH_2_	Propenyl	OH	H	1.0 ± 0.1 (*n* = 3)	0.24 (*n* = 1)
8	**kb-NB123-63**	C=O	H	OH	H	14.9 ± 1.2 (*n* = 3)	0.85 ± 0.11 (*n* = 2)
9	**kb-NB123-89**	CHOH	H	OH	H	24.09 ± 0.71 (*n* = 3)	1.23 ± 0.21 (*n* = 2)
10	**kb-NB142-05**	C=NNHPh	H	OH	H	21.70 ± 0.52 (*n* = 3)	1.13 (*n* = 1)
11	**kb-NB142-11**	C=NNHTs	H	OH	H	38.21 ± 1.17 (*n* = 3)	n.d.
12	**kb-NB142-10**	C=NOBn	H	OH	H	not inhibitory	n.d.

a PKD1 IC_50_ was determined using an automated, HTS formatted IMAP-based PKD Fluorescence Polarization (FP) assay as previously described [52]. Each IC_50_ was calculated as the mean ± SEM of at least three independent experiments with triplicate determinations at each concentration in each experiment; *n* = number of independent experiments.

b PKD1 IC_50_ was determined using a radiometric kinase activity assay as previously described [52]. Each IC_50_ was calculated as the mean ± SEM with triplicate determinations at each concentration in each experiment; *n* = number of independent experiments.

**Table 4.** Chemical structure and PKD1 inhibitory activity of the β-carboline analogs.

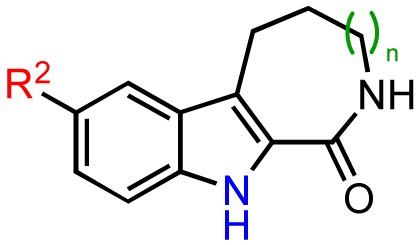						

**Entry**	**Compound**	**Structure**		**IC_50_**		
	
		**R^2^**	**n**	**IMAP-FP PKD1 (μM)**^a^	**Radiometric PKD1 (μM)**^b^	**Cellular PKD1 (μM)**^c^
1	**kb-NB123-59**	OH	0	19.4 ± 1.4 (*n* = 3)	1.57 ± 0.20 (*n* = 2)	n.d.
2	**kb-NB123-57**	OH	1	2.14 ± 0.12 (*n* = 3)	0.13 ± 0.01 (*n* = 3)	>50 (*n* = 3)
3	**kb-NB123-52**	OBn	0	not inhibitory	n.d.	n.d.
4	**kb-NB123-53**	OBn	1	not inhibitory	n.d.	n.d.
5	**kb-NB142-08**	NH_2_	0	74.4 ± 2.2 (*n* = 3)	15.74 ± 0.19 (*n* = 2)	n.d.
6	**kb-NB142-01**	NH_2_	1	47.1 ± 2.5 (*n* = 3)	9.68 ± 1.01 (*n* = 2)	n.d.
7	**kb-NB123-93**	NHAc	0	not inhibitory	n.d.	n.d.
8	**kb-NB123-94**	NHAc	1	not inhibitory	n.d.	n.d.

a PKD1 IC_50_ was determined using an automated, HTS formatted IMAP-based PKD Fluorescence Polarization (FP) assay as previously described [52]. Each IC_50_ was calculated as the mean ± SEM of at least three independent experiments with triplicate determinations at each concentration in each experiment; *n* = number of independent experiments.

b PKD1 IC_50_ was determined using a radiometric kinase activity assay as previously described [52]. Each IC_50_ was calculated as the mean ± SEM of at least two independent experiments with triplicate determinations at each concentration in each experiment; *n* = number of independent experiments.

c Cellular IC_50_ was determined by densitometry analysis of Western blotting data for PKD1 autophosphorylation at S^916^ in LNCaP cells as previously described [53]. Each IC_50_ was calculated as the mean ± SEM of at least three independent experiments; *n* = number of independent experiments.

**Table 5.** Chemical structures and PKD1 inhibitory activities of benzothienothiazepinone analogs.

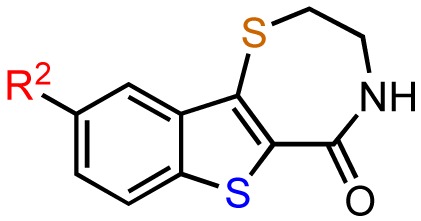						

**Entry**	**Compound**	**Structure**	**IC_50_**		
	
		**R^2^**	**IMAP-FP PKD1 (*μ*M)**^a^	**Radiometric PKD1 (*μ*M)**^b^	**cellular PKD1 (*μ*M)**^c^
1	**kb-NB123-66**	OBn	not inhibitory	n.d.	n.d.
2	**kb-NB142-70**	OH	0.71 ± 0.02 (*n* = 3)	0.028 ± 0.002 (*n* = 3)	2.22 ± 0.59 (*n* = 3)
3	**kb-NB165-09**	OMe	n.d.	0.08 ± 0.01 (*n* = 4)	3.13 ± 0.54 (*n* = 3)

a PKD1 IC_50_ was determined using an automated, HTS formatted IMAP-based PKD Fluorescence Polarization (FP) assay as previously described [52]. Each IC_50_ was calculated as the mean ± SEM of at least three independent experiments with triplicate determinations at each concentration in each experiment; *n* = number of independent experiments.

b PKD1 IC_50_ was determined using a radiometric kinase activity assay as previously described [52]. Each IC_50_ was calculated as the mean ± SEM of at least three independent experiments with triplicate determinations at each concentration in each experiment; *n* = number of independent experiments.

c Cellular IC_50_ was determined by densitometry analysis of Western blotting data for PKD1 autophosphorylation at S^916^ in LNCaP cells as previously described [53]. Each IC_50_ was calculated as the mean ± SEM of at least three independent experiments; *n* = number of independent experiments.

**Table 6.** Chemical structures and PKD1 inhibitory activities of analogs with zone I modifications.

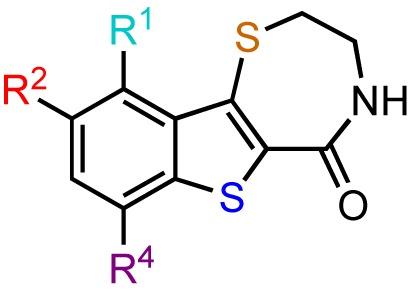							

**Entry**	**Compound**	**Structure**			**IC_50_**		
	
		**R^1^**	**R^2^**	**R^4^**	**% PKD1 activity at 1 *μ*M**	**Radiometric PKD1 (*μ*M)**^a^	**Cellular PKD1 (*μ*M)**^b^
1	**mcf292-03**	H	NH	H	74.4 ± 1.1 (*n* = 2)	3.17 (*n* = 1)	n.d.
2	**mcf292-08**	H	N_3_	H	n.d.	0.08 ± 0.01 (*n* = 5)	2.17 ± 0.22 (*n* = 3)
3	**mcf292-05**	H	N=C=S	H	n.d.	2.77 (*n* = 1)	n.d.
4	**mcf292-09**	H	NHCOCH_2_Cl	H	n.d.	1.50 (*n* = 1)	n.d.
5	**kb-NB165-31**	I	OH	H	13.6 (*n* = 1)	0.11 ± 0.02 (*n* = 3)	8.6 ± 2.0 (*n* = 3)
6	**kb-NB184-52**	Br	OH	H	12.7 ± 0.2 (*n* = 2)	0.048 (*n* = 1)	n.d.
7	**kb-NB184-38**	H	H	OBn	98.6 ± 4.1 (*n* = 2)	n.d.	n.d.
8	**kb-NB184-40**	H	H	OH	99 ± 11 (*n* = 2)	n.d.	n.d.
9	**kb-NB184-44**	H	H	OMe	77.5 ± 3.6 (*n* = 2)	n.d.	n.d.

a PKD1 IC_50_ was determined using a radiometric kinase activity assay as previously described [52]. Each IC_50_ was calculated as the mean ± SEM with triplicate determinations at each concentration in each experiment; *n* = number of independent experiments.

b Cellular IC_50_ was determined by densitometry analysis of Western blotting data for PKD1 autophosphorylation at S^916^ in LNCaP cells as previously described [53]. Each IC_50_ was calculated as the mean ± SEM of at least three independent experiments; *n* = number of independent experiments.

**Table 7.** Chemical structures and PKD1 inhibitory activities of zone II and III modifications.

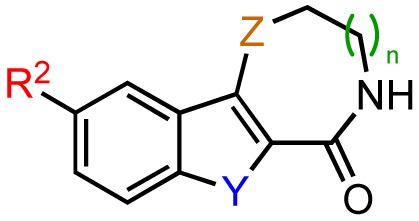								

**Entry**	**Compound**	**Structure**				**IC_50_**		
	
		**Y**	**Z**	**R^2^**	**n**	**% PKD1 activity at 1 μM**	**Radiometric PKD1 (μM)**^a^	**Cellular PKD1 (μM)**^b^
1	**kb-NB184-22**	S=O	S	OH	1	66.5 ± 6.1 (*n* = 2)	n.d.	n.d.
2	**kb-NB184-25**	S=O	S	OMe	1	50.4 ± 2.3 (*n* = 2)	1.08 (*n* = 1)	n.d.
3	**kb-NB184-45**	S	S=O	OMe	1	97 ± 16 (*n* = 2)	n.d.	n.d.
4	**kb-NB165-89**	S	S	OBn	2	84.3 (*n* = 1)	21.7 (*n* = 1)	n.d.
5	**kb-NB165-92**	S	S	OH	2	16.7 (*n* = 1)	0.11 ± 0.01 (*n* = 3)	2.56 ± 0.66 (*n* = 2)
6	**kb-NB184-02**	S	S	OMe	2	29.5 (*n* = 1)	0.19 ± 0.03 (*n* = 3)	18.6 ± 2.0 (*n* = 3)
7	**kb-NB184-36**	S	O	OBn	2	83.3 ± 3.8 (*n* = 2)	n.d.	n.d.
8	**kb-NB184-57**	S	O	OMe	2	62.0 ± 3.5 (*n* = 2)	n.d.	n.d.
9	**kb-NB184-80**	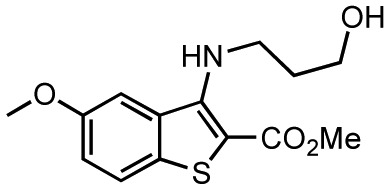				91.3 ± 1.5 (*n* = 2)	not inhibitory	n.d.

a PKD1 IC_50_ was determined using a radiometric kinase activity assay as previously described [52]. Each IC_50_ was calculated as the mean ± SEM with triplicate determinations at each concentration in each experiment; *n* = number of independent experiments.

b Cellular IC_50_ was determined by densitometry analysis of Western blotting data for PKD1 autophosphorylation at S^916^ in LNCaP cells as previously described [53]. Each IC_50_ was calculated as the mean ± SEM of at least two independent experiments; *n* = number of independent experiments.

**Table 8.** Chemical structures and PKD1 inhibitory activities of zone IV modifications.

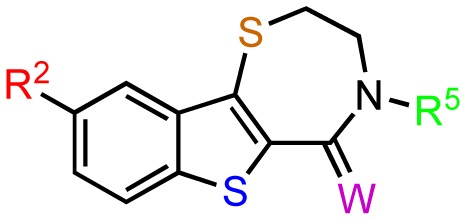							

**Entry**	**Compound**	**Structure**			**IC^50^**	
	
		**W**	**R^2^**	**R^5^**	**% PKD1 activity at 1 *μ*M**	**Radiometric PKD1 (*μ* M)**^a^
1	**kb-NB165-16**	O	OMe	Me		4.57 ± 0.78 (*n = 2*)
2	**kb-NB165-17**	O	OH	Me		0.45 ± 0.05 (*n = 2*)
3	**kb-NB165-75**	O	OH	(CH_2_)_2_NH_2_	55.6 (*n* = 1)	0.757 (*n* = 1)
4	**kb-NB165-81**	--	OBn	H	78.3 (*n* = 1)	39.6 (*n* = 1)
5	**kb-NB165-83**	--	OH	H	92.4 (*n* = 1)	16.4 (*n* = 1)

a PKD1 IC_50_ was determined using a radiometric kinase activity assay as previously described [52]. Each IC_50_ was calculated as the mean ± SEM with triplicate determinations at each concentration in each experiment; *n* = number of independent experiments.

**Table 9.** Chemical structures and PKD1 inhibitory activities of analogs with zone I modifications to the pyrimidine scaffold.

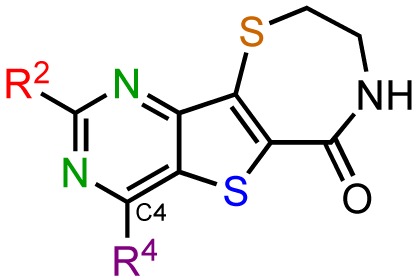					

**Entry**	**Compound**	**Structure**		**IC_50_**	
	
		**R^2^**	**R^4^**	**Radiometric PKD1 (*μ*M)**^a^	**Cellular PKD1 (*μ* M)**^b^
1	**kmg-NB4-23**	OMe	H	0.12 ± 0.03 (*n* = 4)	6.8 ± 1.3 (*n* = 3)
2	**kmg-NB4-69A**	OH	H	25.3 (*n* = 1)	n.d.
3	**kmg-NB5-13C**	OMe	OMe	>30.0 (*n* = 2)	n.d.
4	**kmg-NB5-15A**	OMe	OH	>30.0 (*n* = 2)	n.d.

a PKD1 IC_50_ was determined using a radiometric kinase activity assay as previously described [52]. Each IC_50_ was calculated as the mean ± SEM with triplicate determinations at each concentration in each experiment; *n* = number of independent experiments.

b Cellular IC_50_ was determined by densitometry analysis of Western blotting data for PKD1 autophosphorylation at S^916^ in LNCaP cells as previously described [53]. Each IC_50_ was calculated as the mean ± SEM of at least three independent experiments; *n* = number of independent experiments.

**Table 10.** Functionalizations of **CID755673**.

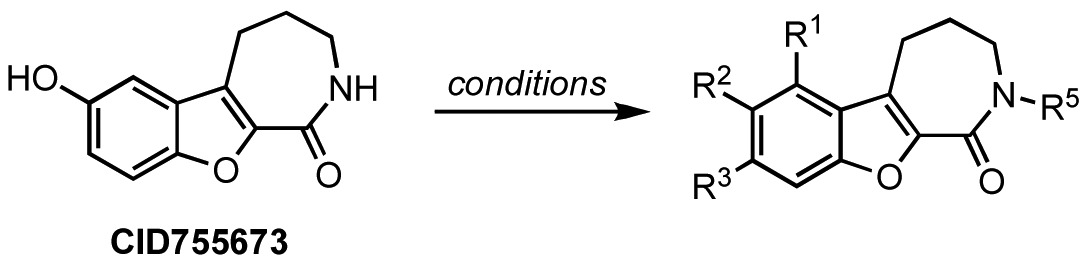							

**Entry**	**Conditions**	**Structure**				**Yield, %**	**Product**

		**R^1^**	**R^2^**	**R^3^**	**R^5^**		
1	MeI, K_2_CO_3_	H	OMe	H	H	74	**kb-NB77-56**
2	AllylBr, K_2_CO_3_	H	OAllyl	H	H	69	**kb-NB77-84**
3	TBSCl, *i*-Pr_2_NEt	H	OTBS	H	H	91	**kb-NB77-77**
4	AcCl (2 equiv), DMAP	H	OAc	H	H	91	**kb-NB123-36**
5	MeI, KO*t-*Bu	H	OMe	H	Me	34	**kb-NB96-04**
6	AcCl (3 equiv), DMAP	H	OAc	H	Ac	33	**kb-NB123-45-1**
7	*N*-Chlorosuccinimide (1.05 equiv)	Cl	OH	H	H	86	**kb-NB77-88**
8	Selectflour^®^	F	OH	H	H	29	**kb-NB96-21**
9	*N*-Chlorosuccinimide (2 equiv)	Cl	OH	Cl	H	73	**kb-NB96-43**

**Table 11.** Zone I modifications based on **mcf292-03**.

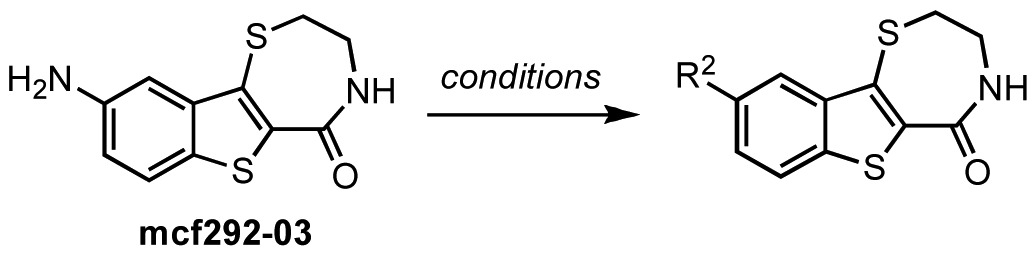				

**Entry**	**Condition**	**Structure**	**Yield, % (from 21)**	**Product**

		**R^2^**		
1	*t*BuONO, TMSN_3_, MeCN, rt	N_3_	38%	**mcf292-08**
2	CSCl_2_, NaHCO_3_, CHCl_3_/H_2_O, rt	NCS	41%	**mcf292-05**
3	ClCH_2_COCl, 2,6-lutidine, DCM, rt	NHCOCH_2_Cl	46%	**mcf292-09**
